# *Lacticaseibacillus rhamnosus* CRL1505 Enhances Antiviral State in Respiratory Epithelial Cells and Alveolar Macrophages

**DOI:** 10.3390/biology15211927

**Published:** 2026-10-23

**Authors:** Luciano Arellano-Arriagada, Ayelen Antonella Baillo, Leonardo Albarracin, Solange Cisterna-Vergara, Xi-Chen Bai, Wenxiao Du, Weichen Gong, Yoshihito Suda, Haruki Kitazawa, Julio Villena

**Affiliations:** 1 Laboratory of Respiratory Immunology (LaRI), Division of Animal Immunology and Omics, International Education and Research Center for Food and Agricultural Immunology (CFAI), Graduate School of Agricultural Science, Tohoku University, Sendai 980-8572, Japanalbarracin.leonardo.miguel.e5@tohoku.ac.jp (L.A.); bai.xichen.r4@dc.tohoku.ac.jp (X.-C.B.); du.wenxiao.t5@dc.tohoku.ac.jp (W.D.); 2 Food and Feed Immunology Group, Laboratory of Animal Food Function, Graduate School of Agricultural Science, Tohoku University, Sendai 980-8572, Japan; gong.weichen.c1@tohoku.ac.jp; 3 Laboratory of Immunobiotechnology, Reference Centre for Lactobacilli (CERELA-CONICET), Tucuman T4000, Argentina; abaillo@cerela.org.ar; 4 Livestock Immunology Unit, International Education and Research Centre for Food and Agricultural Immunology (CFAI), Graduate School of Agricultural Science, Tohoku University, Sendai 980-8572, Japan; 5 Department of Food, Agriculture and Environment, Miyagi University, Sendai 980-8572, Japan; suda@myu.ac.jp

**Keywords:** *L. rhamnosus* CRL1505, respiratory epithelial cells, alveolar macrophages, SIV, PRRSV, antiviral immunity, immunobiotics

## Abstract

Respiratory viral infections remain a major health concern, highlighting the need for strategies that enhance host antiviral defenses. *Lacticaseibacillus rhamnosus* CRL1505 has demonstrated beneficial immunomodulatory effects in the respiratory tract; however, its direct effects on distinct respiratory cell populations remain incompletely characterized. Here, we evaluated the capacity of live and HK 1505 to modulate antiviral immunity in porcine bronchial epithelial (PBE) cells challenged with SIV and porcine alveolar macrophage (PAM) cells infected with PRRSV. Pretreatment with either live or HK 1505 significantly reduced infectious SIV titers in PBE cells and decreased PRRSV ORF5 expression and viral antigen detection in PAM cells, accompanied by enhanced expression of interferons and antiviral factors in both models. CRL1505 also differentially modulated inflammatory responses according to the cell type. In PAM cells, both treatments enhanced proinflammatory and regulatory cytokines, whereas in PBE cells they selectively increased IL-1β and IL-6 while reducing TNF-α, IL-8, and MCP-1. Negative regulators of innate immune signaling were also differentially modulated in both cell types. Importantly, HK 1505 retained significant antiviral and immunomodulatory activities, inducing responses that were generally similar to those observed with the viable preparation under the experimental conditions evaluated. These findings demonstrate that *L. rhamnosus* CRL1505 enhances antiviral responses in two distinct respiratory cell models, with differences in immunomodulatory profiles that may reflect the combined influence of cellular and viral contexts.

## Simple Summary

Respiratory viral infections remain an important health concern, highlighting the need for new strategies that strengthen the natural defenses of the respiratory tract. *Lacticaseibacillus rhamnosus* CRL1505 is an immunobiotic bacterium with a demonstrated ability to beneficially modulate respiratory immunity. In this study, we investigated whether live and heat-killed (HK) CRL1505 (HK 1505) could improve antiviral responses in two important respiratory cell populations: respiratory epithelial cells (RECs) and alveolar macrophages. Swine influenza virus (SIV) and porcine reproductive and respiratory syndrome virus (PRRSV) were used as viral challenges. Both live and HK 1505 reduced viral infection and enhanced cellular antiviral defenses. In addition, CRL1505 differentially modulated inflammatory and regulatory responses depending on the respiratory cell type, indicating that its effects extend beyond the induction of antiviral factors. Importantly, HK 1505 retained the beneficial effects observed with the live bacterium. These findings demonstrate that CRL1505 can enhance antiviral immunity in distinct respiratory cell populations and support further investigation of non-viable CRL1505 preparations as a potential strategy to improve resistance to respiratory viral infections.

## 1. Introduction

Respiratory viral infections represent a major global health burden, causing substantial morbidity and mortality worldwide, particularly among vulnerable populations such as infants, older adults, and immunocompromised individuals. A wide variety of viruses can infect the respiratory tract, including influenza viruses, respiratory syncytial virus (RSV), coronaviruses, rhinoviruses, and human metapneumovirus, and their circulation and interactions can influence viral replication and disease severity. Moreover, respiratory viral infections may extend beyond the respiratory tract and, in some cases, be associated with systemic viral dissemination and more severe clinical outcomes [1,2,3]. Despite important advances in vaccines and antiviral therapies, effective preventive and therapeutic options remain limited for several respiratory viruses, highlighting the need for complementary strategies capable of improving host resistance to infection.

Increasing attention has been directed toward strategies capable of strengthening local innate immune defenses rather than acting directly on respiratory pathogens. In this context, beneficial microorganisms with immunomodulatory properties, commonly referred to as immunobiotics, have emerged as promising tools for enhancing resistance to respiratory infections [4]. Their beneficial effects can involve the modulation of epithelial and immune cell functions, resulting in improved antiviral defenses while contributing to the regulation of inflammatory responses [5]. Moreover, these effects are highly strain-dependent, emphasizing the need for a detailed characterization of individual immunobiotic strains and their mechanisms of action. Studies using viable microorganisms, non-viable cells, bacterial components, and cell-free products have demonstrated that modulation of respiratory immunity can be achieved through different microbial-derived stimuli, further supporting the potential of this approach for controlling respiratory infections [6,7].

Among the immunobiotic strains investigated in this context, *Lacticaseibacillus rhamnosus* CRL1505 has been extensively characterized for its capacity to beneficially modulate respiratory immunity. Early studies demonstrated that nasal administration of CRL1505 improved the respiratory antiviral immune response triggered by Toll-like receptor (TLR)-3 activation and increased resistance to RSV infection [7,8]. Importantly, comparison with other *L. rhamnosus* strains revealed that these effects were strain-dependent, indicating that the antiviral activity of CRL1505 represents a specific functional property rather than a general characteristic of lactobacilli [9]. Notably, the immunomodulatory activity of CRL1505 does not strictly depend on bacterial viability. The peptidoglycan purified from CRL1505 retains the capacity to modulate respiratory antiviral immunity and improves resistance to primary RSV infection and secondary pneumococcal pneumonia [10]. This protection was associated with enhanced antiviral and regulatory responses, including increased interferon (IFN)-β, IFN-γ, and interleukin (IL)-10 in the respiratory tract. Subsequent studies identified alveolar macrophages as important cellular mediators of these effects. Non-viable CRL1505 and its peptidoglycan modulated alveolar macrophage responses, and macrophage depletion experiments demonstrated the contribution of these cells to improved pathogen control and protection against lung injury during RSV infection and pneumococcal superinfection [11].

Despite these advances, the direct effects of *L. rhamnosus* CRL1505 on distinct cellular components of the respiratory antiviral immune system remain incompletely characterized. In particular, it remains to be determined whether CRL1505 can enhance antiviral defenses in both RECs and alveolar macrophages, whether these effects are maintained against viruses with distinct cellular tropisms, and to what extent bacterial viability is required for its antiviral activity. To address these questions, we used two in vitro respiratory infection models: SIV infection in PBE cells [12] and PRRSV infection in PAM cells [13]. The selection of porcine respiratory cell models was based on the availability of previously established and well-characterized experimental systems that allow the investigation of antiviral immune responses in distinct respiratory cell populations. In particular, the different cellular tropisms of SIV and PRRSV provide complementary models for evaluating the immunomodulatory effects of CRL1505 in RECs and alveolar macrophages, respectively. Although these models do not fully reproduce human respiratory immunity, they provide a valuable experimental approach for investigating cellular mechanisms that may be relevant to respiratory antiviral defense across species. We investigated the capacity of viable and HK 1505 to enhance resistance to viral infection and characterized its effects on IFNs, antiviral factors, inflammatory and regulatory mediators, and negative regulators of innate immune signaling. By using these in vitro models, we aim to better understand how CRL1505 influences antiviral and inflammatory responses at the cellular level across distinct respiratory cell populations.

## 2. Materials and Methods

### 2.1. Porcine Respiratory Cell Lines

The PAM-KNU-hTERT porcine alveolar macrophage cell line (Cat. No. T0741-C, ABM, Richmond, BC, Canada), originally established from lung tissue of a euthanized 8-week-old specific pathogen-free (SPF) pig, was used in this study. PAM cells were maintained in tissue culture flasks (Corning, NY, USA) using Roswell Park Memorial Institute (RPMI)-1640 medium (FUJIFILM Wako Pure Chemical Corporation, Tokyo, Japan) supplemented with 10% fetal calf serum (FCS) and 1% penicillin–streptomycin solution. Cells were cultured at 37 °C in a humidified atmosphere containing 5% CO_2_ [13].

The PBE cell line was previously established from bronchial tissue obtained from a 7-day-old piglet as described in [14]. Cells were maintained in DMEM (GIBCO, Grand Island, NY, USA) supplemented with 10% FCS and 1% penicillin/streptomycin. Cultures were incubated at 37 °C under a humidified atmosphere containing 5% CO_2_ using a CO_2_ incubator (Thermo Fisher Scientific, Waltham, MA, USA).

### 2.2. Porcine Respiratory Viruses

The PRRSV EDRD-1 strain and the SIV H1N1 subtype (A/swine/Tottori/41-6255/2018) were obtained from the National Agriculture and Food Research Organization (Tsukuba, Japan).

PRRSV viral stocks were prepared by propagation in the PAM-KNU cell line, divided into aliquots, and maintained at −80 °C until required for experiments. PRRSV titers were determined according to the previously described procedure [15].

SIV H1N1 was propagated in Madin–Darby canine kidney cells (MDCKs). Viral stocks were titrated on MDCK cells. For viral titration, SIV was serially diluted in serum-free DMEM (GIBCO, NY, USA) containing 1 µg/mL tosylsulfonyl phenylalanyl chloromethyl ketone (TPCK)-trypsin (Sigma, St. Louis, MO, USA). Aliquots of 100 µL from each dilution were added to MDCK cell monolayers cultured in 96-well plates and incubated for 168 h. Viral titers were subsequently determined as the 50% tissue culture infectious dose (TCID_50_) according to the Reed–Muench method as described previously [12].

### 2.3. Preparation of Viable and Heat-Killed Lacticaseibacillus rhamnosus CRL1505

*Lacticaseibacillus rhamnosus* CRL1505 was obtained from the culture collection of CERELA-CONICET (San Miguel de Tucumán, Argentina). The strain was initially streaked onto de Man, Rogosa and Sharpe (MRS) agar (Sigma, Detroit, MI, USA) and cultured for 24 h. Following incubation, a single colony was selected and inoculated into 1 mL of MRS broth, where it was cultured for an additional 16 h before use in subsequent experimental procedures.

For the preparation of HK 1505, bacterial cells obtained after cultivation were harvested by centrifugation, washed with sterile phosphate-buffered saline (PBS), and resuspended in PBS. The bacterial suspension was then heat-treated at 90 °C for 30 min. After treatment, the suspension was cooled to room temperature and used for subsequent experiments. The absence of bacterial viability was confirmed by plating an aliquot onto MRS agar and verifying the absence of colony growth after incubation.

The bacterial concentration was initially determined by colony-forming unit (CFU) enumeration before heat treatment. Following thermal inactivation, the bacterial cell concentration was verified by direct microscopic counting using a Neubauer counting chamber. Viable and HK 1505 bacterial suspensions were standardized to equivalent bacterial cell numbers, and the multiplicity of infection (MOI) of 10 was calculated based on the ratio of bacterial cells to host cells.

### 2.4. Treatment of Respiratory Cell Lines with Lactobacilli and Viral Pathogens

PAM cells were seeded in 24-well tissue culture plates at a density of 1 × 10^5^ cells/well and cultured for 24 h. The cells were subsequently stimulated with viable or HK 1505 at an MOI of 10 and incubated for an additional 12 h at 37 °C in an atmosphere containing 5% CO_2_. Following bacterial stimulation, the bacterial suspensions were removed, and the cells were washed with medium to eliminate residual bacteria before viral challenge. PAM cells were challenged with PRRSV at an MOI of 0.05.

PBE cells were seeded in 24-well tissue culture plates at a density of 1 × 10^4^ cells/well. The cells were then stimulated with viable or HK 1505 at an MOI of 10 and maintained at 37 °C in an atmosphere containing 5% CO_2_ for 24 h. Following bacterial stimulation, the bacterial suspensions were removed, and the cells were washed with medium to eliminate residual bacteria before viral challenge. PBE cells were exposed to SIV at an MOI of 0.01 in the presence of 1 µg/mL TPCK-trypsin for 1 h. Mock-infected control cells were incubated for the same period with virus-free culture medium containing 1 µg/mL TPCK-trypsin. Following viral exposure, the inoculum was removed, and the cells were washed twice with culture medium prewarmed to 37 °C. The cells were then maintained in culture medium containing TPCK-trypsin.

The different seeding densities were selected according to the growth characteristics and previously established culture conditions for each cell line. Both PAM and PBE cells reached more than 90% confluence before viral challenge.

The pretreatment periods (12 h for PAM cells and 24 h for PBE cells) were selected based on preliminary experiments evaluating cell viability following exposure to viable CRL1505, allowing the identification of suitable incubation conditions for each cellular model.

### 2.5. Real-Time Quantitative PCR

Total RNA was isolated from PBE cells and PAM cells collected at the specified time points following viral challenges using TRIZOL reagent (Invitrogen, Carlsbad, CA, USA), following the manufacturer’s protocol. RNA concentration was determined from absorbance measurements at 260 nm using a NanoDrop^®^ ND-1000 Spectrophotometer (Thermo Fisher Scientific, MA, USA), and sample purity was assessed based on the A260/A280 ratio, which ranged from 1.8 to 2.0 for all samples. RNA was subsequently reverse-transcribed using the PrimeScript RT Reagent Kit (Takara Bio, Shiga, Japan) according to the manufacturer’s instructions.

Quantitative real-time PCR was performed with TB Green Premix Ex Taq (Takara Bio, Shiga, Japan) on a CFX Connect Real-Time PCR System (Bio-Rad, Hercules, CA, USA). The amplification program consisted of an initial step at 95 °C for 30 s, followed by 40 cycles of 95 °C for 5 s and 60 °C for 30 s. The primer sequences used for amplification have been described previously [12,13]. The study included the evaluation of the viral genes ORF5 for PRRSV, NP for SIV, and immune factors: IFN-β, IFN-γ, IFN-λ1, IFN-λ3, Mx1, Mx2, OAS1, OAS2, OASL, RNAse L, IL-10, IL-27, TNF-α, IL-1β, IL-6, IL-8, MCP-1, A20, BCL-3, Tollip, SIGIRR, IRAK-M, and MKP-1.

Based on our previous study [14], β-actin was selected as the housekeeping gene because of its stable expression across different porcine tissues. Transcript levels were quantified using a standard curve generated from serial dilutions of plasmid DNA. For each sample, the expression level of the target gene was normalized to that of β-actin, and the resulting values were expressed relative to the control group, which was assigned a value of 1.

### 2.6. Immunofluorescence

PAM cells were washed once with PBS and fixed with 80% acetone for 5 min at 4 °C. After fixation, the acetone was removed and the plates were allowed to air-dry for 1 h. The cells were then incubated for 1 h with the anti-PRRSV monoclonal antibody SR30A (RTI, LLC), diluted 1:1000 in blocking buffer consisting of 1% goat serum in PBS. Following five washes with PBS, Alexa Fluor 488-conjugated Affini Pure anti-mouse IgG (Sigma-Aldrich, Burlington, MA, USA) was added and incubated for 1 h. The wells were subsequently washed five times with PBS, and 1–2 drops of DAPI were added for nuclear staining. Fluorescence images were acquired using a BZ-X800 All-in-One Fluorescence Microscope (KEYENCE, Osaka, Japan).

### 2.7. Scanning Electron Microscopy (SEM)

To evaluate the interaction of *L. rhamnosus* CRL1505 with PAM cells, the cells were cultured on inserts and coincubated with either live or HK 1505. After 2 h of coincubation, the samples were fixed in 2% (*v*/*v*) glutaraldehyde for 1 h. The inserts were subsequently dehydrated by sequential immersion in 50, 60, 70, 80, 90, and 99% ethanol for 20 min at each concentration. Following dehydration, the samples were immersed in *t*-butyl alcohol, lyophilized, and coated with platinum-palladium. The interaction between CRL1505 and PAM cells, including bacterial attachment and apparent engulfment, was examined using a HITACHI scanning electron microscope available at the Graduate School of Agricultural Science, Tohoku University (Sendai, Japan).

### 2.8. Flow Cytometry Analysis of Phagocytosis

Bacterial uptake by PAM cells was evaluated by flow cytometry as previously described [16], with minor modifications. Briefly, PAM cells were incubated with fluorescently labeled viable or HK 1505. Following incubation, cells were washed with PBS, harvested, and analyzed by flow cytometry. The interaction between bacteria and PAM cells was quantified based on mean fluorescence intensity (MFI). Heat-killed *Lactobacillus gasseri* 36 (HK 36) was included as a control because of its lower capacity to be phagocyted by PAM cells [16].

### 2.9. Statistical Analysis

Statistical analyses were conducted using GraphPad Prism version 8 (GraphPad Software, San Diego, CA, USA). After verifying that the data followed a normal distribution, differences among experimental groups were analyzed by two-way analysis of variance (ANOVA), followed by Tukey’s multiple-comparison test for pairwise comparisons between group means. Differences were considered statistically significant when *p* < 0.05. All experiments were performed independently three times (n = 3), representing three independent biological replicates. Data are presented as the mean ± standard deviation (SD).

## 3. Results

### 3.1. Lacticaseibacillus rhamnosus CRL1505 Modulates Cytokine Profile in Porcine Alveolar Macrophages and Respiratory Epithelial Cells

**Figure 1:**
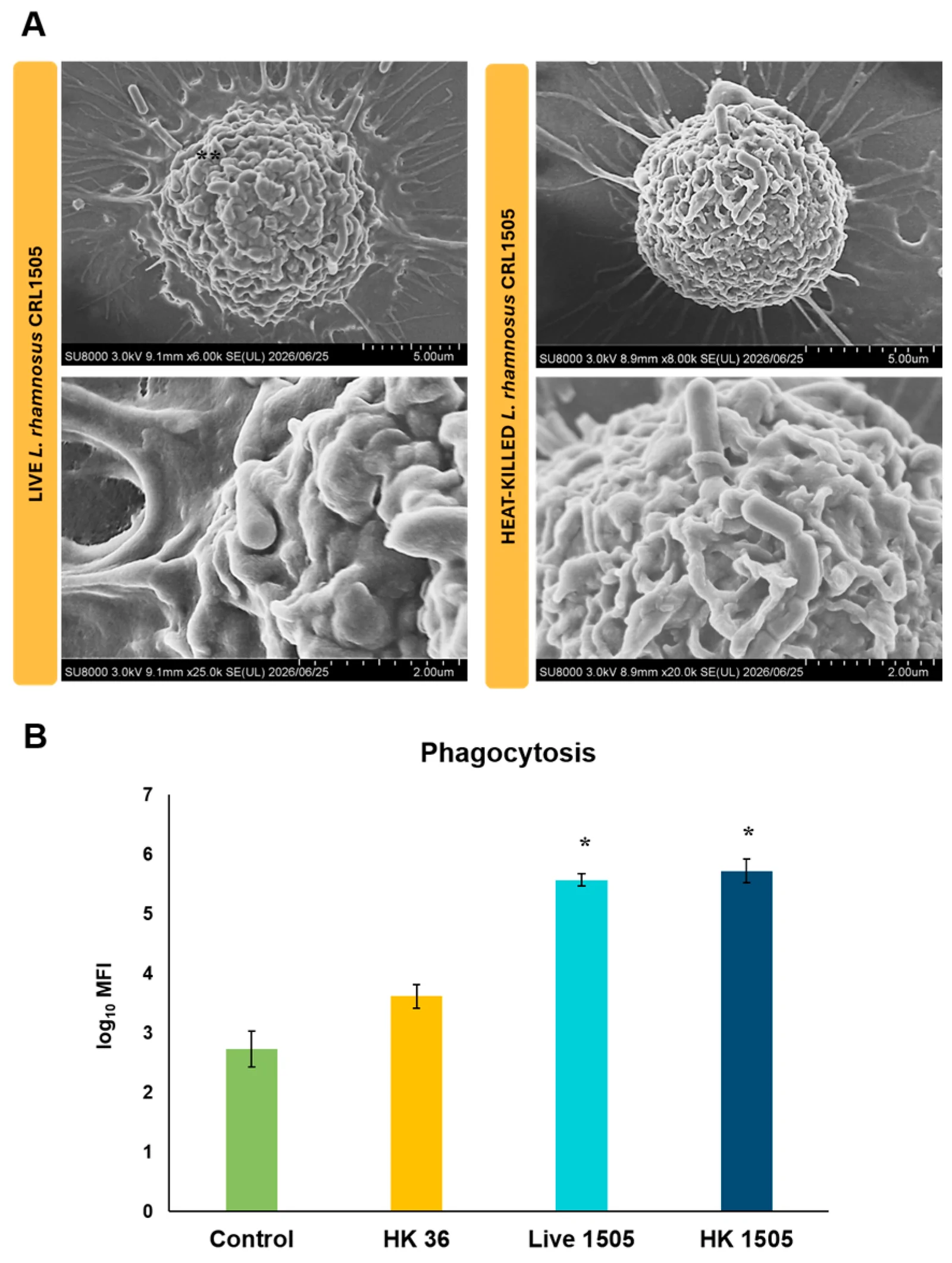
(**A**) Scanning electron microscopy analysis of the interaction between live and heat-killed *Lacticaseibacillus rhamnosus* CRL1505 and porcine alveolar macrophages (PAM cells). PAM cells were co-incubated with viable (Live 1505) or heat-killed (HK 1505) *L. rhamnosus* CRL1505 for 2 h prior to fixation and SEM processing. Representative micrographs of PAM cells co-cultured with lactobacilli, showing extensive pseudopod extensions, filopodial projections, and phagocytic cup formation around individual bacteria. Membrane ruffling consistent with active actin cytoskeleton remodeling and bacterial engulfment is evident. (**B**) Phagocytosis of live and HK 1505 by PAM cells. Alveolar macrophages were stimulated with live or HK 1505, and phagocytosis was evaluated by flow cytometry. Heat-killed *Lactobacillus gasseri* 36 (HK 36) was included as a control because of its lower capacity to be phagocyted by PAM cells. The results are expressed as log_10_ mean fluorescence intensity (MFI) and shown as mean ± SD from three independent biological experiments (n = 3). Significant differences are shown compared to the control group at *p* < 0.05 (*).

We previously demonstrated that lactobacilli strains more efficiently phagocytosed by alveolar macrophages have a greater ability to improve antiviral immune responses through the modulation of TLR3 activation [16]. Therefore, we first evaluated whether there were differences in the phagocytosis of live and HK 1505 by porcine alveolar macrophages (PAM cells). SEM analysis showed that both live and HK 1505 closely interacted with PAM cells and were observed at different stages of apparent engulfment (Figure 1A). Bacterial cells were frequently associated with the macrophage surface, together with pseudopod extension and membrane structures consistent with bacterial internalization. No evident qualitative differences were observed between the two preparations under the conditions evaluated. In addition, as shown in Figure 1B, both live and HK 1505 were efficiently phagocyted by PAM cells as determined by flow cytometry. Similar MFI values were observed when live and HK 1505 were compared.

The effect of both live and HK 1505 on PAM cells cytokine expression was also evaluated (Supplementary Figure S1). Both treatments increased IFN-β and IFN-γ expression in PAM cells, with a more pronounced effect on IFN-γ. Similarly, both live and HK 1505 significantly enhanced the expression of TNF-α, IL-1β, and IL-6 compared with control cells, whereas no significant changes were observed in MCP-1 or IL-8 expression. In addition, both treatments increased the expression of the regulatory cytokine IL-10 (Supplementary Figure S1), with no significant differences between the viable and HK preparations. In contrast, neither treatment significantly affected IL-27 expression. In addition, the effect of live and HK 1505 on PBE cells cytokine expression was evaluated (Supplementary Figure S2). The expression levels of IFN-β, IFN-λ1, and IFN-λ3 were significantly higher in PBE cells treated with either live or HK 1505 compared with control cells, with no significant differences between the two treatments. Similarly, both preparations significantly increased IL-1β and IL-6 expression, whereas TNF-α, MCP-1, and IL-8 expression levels remained comparable to those observed in control cells (Supplementary Figure S2).

These results indicate that both live and HK 1505 can interact with alveolar macrophages and RECs, inducing transcriptional changes in several immune factors relevant to innate antiviral immunity.

### 3.2. Lacticaseibacillus rhamnosus CRL1505 Improves Resistance of Porcine Alveolar Macrophages to PRRSV Infection

**Figure 2:**
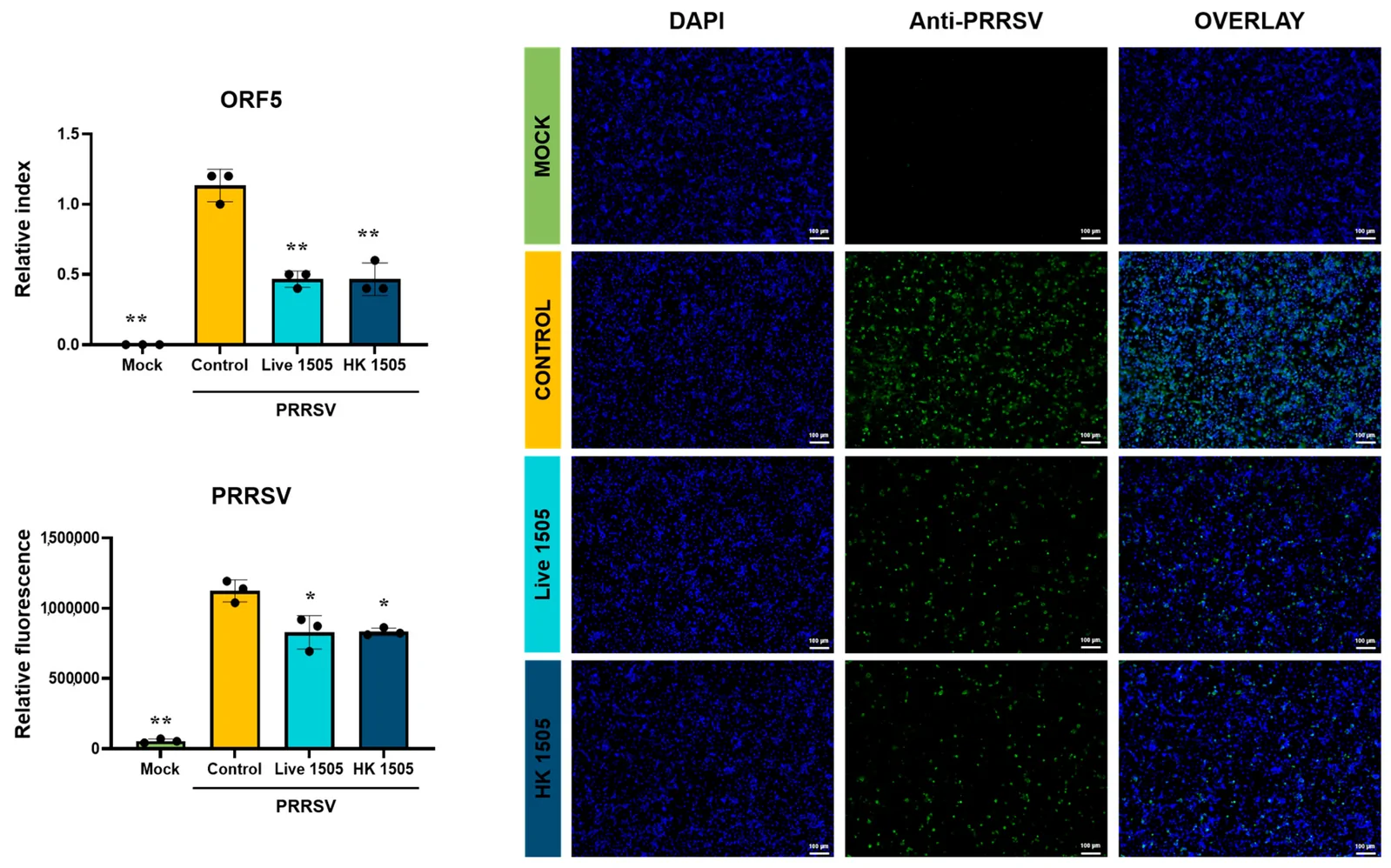
Effect of *Lacticaseibacillus rhamnosus* CRL1505 on PRRSV replication in porcine alveolar macrophages. Porcine alveolar macrophages (PAM cells) were stimulated with viable (Live 1505) or heat-killed (HK 1505) *L. rhamnosus* CRL1505 for 12 h (MOI = 10) and subsequently infected with PRRSV (MOI = 0.05). PRRSV-infected control cells (Control), and non-lactobacilli treated and non-infected PAM cells (Mock) were used for comparisons. Viral replication was assessed at 24 h post-infection by measuring the relative expression of PRRSV ORF5 gene using RT-qPCR and by immunofluorescence assay (IFA) targeting PRRSV antigen. Representative images showing PRRSV antigen-positive cells and DAPI-stained nuclei in each experimental group are shown. White lines indicate 100 µm. The results are shown as mean ± SD from three independent biological experiments (n = 3). Significant differences are shown compared to the control group at *p* < 0.05 (*) or *p* < 0.01 (**).

We next evaluated whether the pretreatment of PAM cells with live or HK 1505 could affect the resistance of macrophages to PRRSV infection (Figure 2). As we demonstrated recently [13], PAM cells can be efficiently infected with PRRSV as confirmed by RT-qPCR detection of the viral ORF5 gene and immunofluorescence detection of viral antigens. Of note, when PAM cells were pretreated with either live or HK 1505, both ORF5 expression and viral antigens were significantly lower than infected control macrophages (Figure 2). Furthermore, no significant differences were found when live- and HK 1505-treated cells were compared.

### 3.3. Lacticaseibacillus rhamnosus CRL1505 Modulates Immune Response to PRRSV in Porcine Alveolar Macrophages

**Figure 3:**
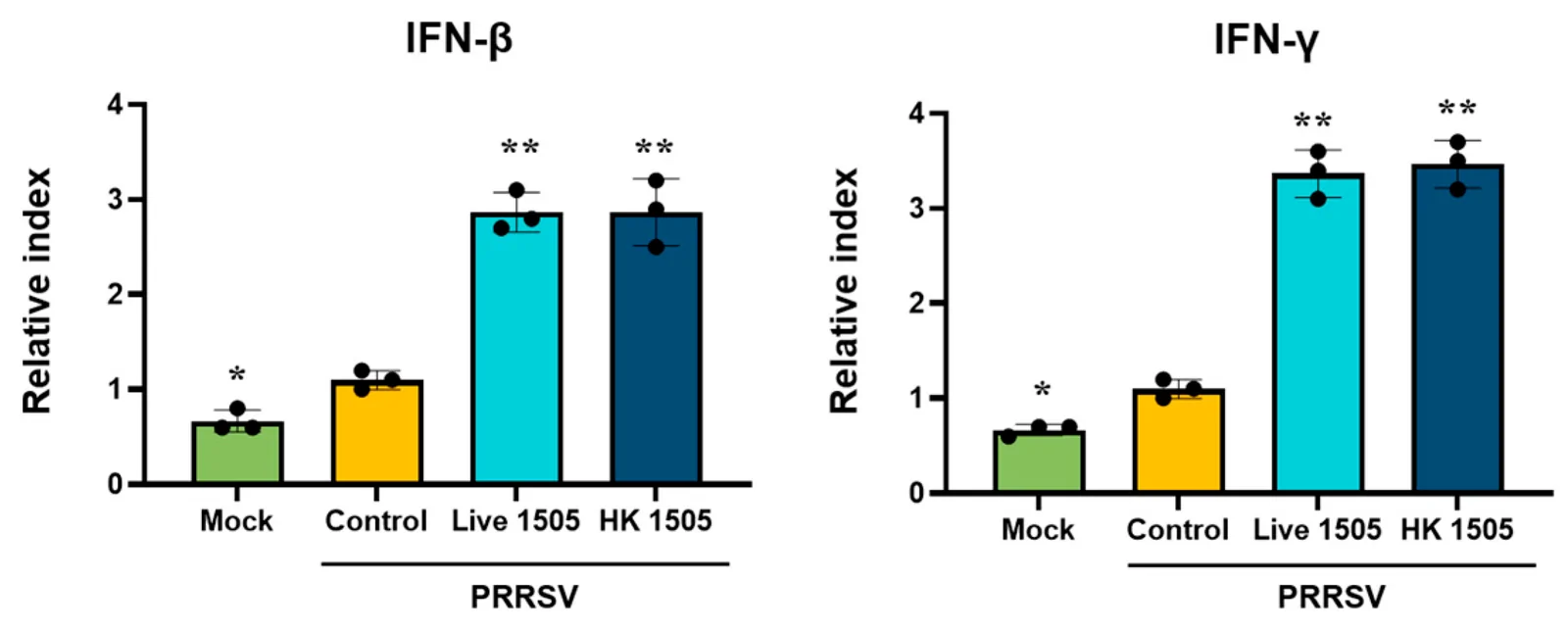
Effect of *Lacticaseibacillus rhamnosus* CRL1505 on the expression of interferons in porcine alveolar macrophages after PRRSV infection. Porcine alveolar macrophages (PAM cells) were stimulated with viable (Live 1505) or heat-killed (HK 1505) *L. rhamnosus* CRL1505 for 12 h (MOI = 10) and subsequently infected with PRRSV (MOI = 0.05). PRRSV-infected control cells (Control), and non-lactobacilli treated and non-infected PAM cells (Mock) were used for comparisons. The expressions of IFN-β and IFN-γ were assessed at 12 h post-infection using RT-qPCR. The results are shown as mean ± SD from three independent biological experiments (n = 3). Significant differences are shown compared to the control group at *p* < 0.05 (*) or *p* < 0.01 (**).

**Figure 4:**
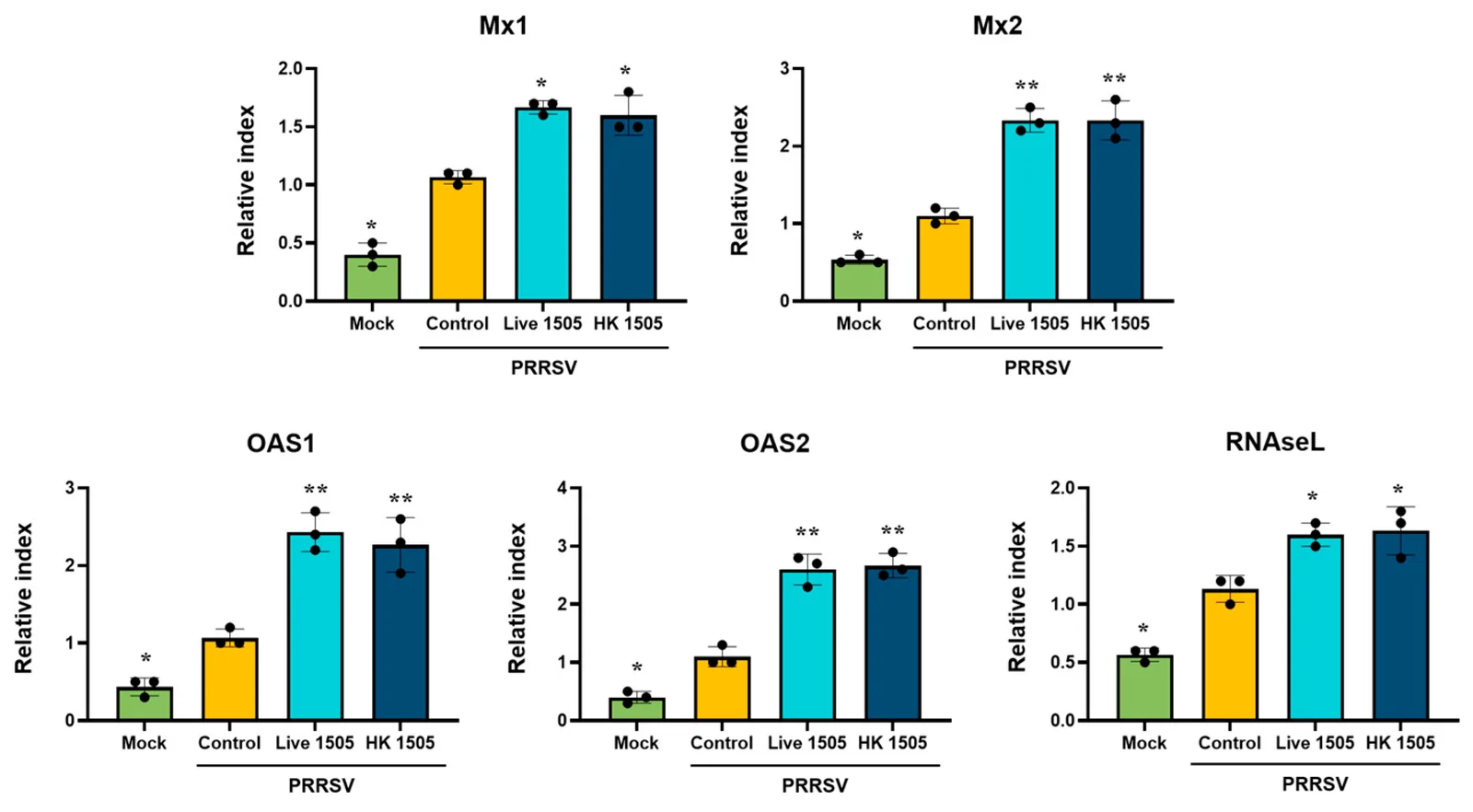
Effect of *Lacticaseibacillus rhamnosus* CRL1505 on the expression of antiviral factors in porcine alveolar macrophages after PRRSV infection. Porcine alveolar macrophages (PAM cells) were stimulated with viable (Live 1505) or heat-killed (HK 1505) *L. rhamnosus* CRL1505 for 12 h (MOI = 10) and subsequently infected with PRRSV (MOI = 0.05). PRRSV-infected control cells (Control), and non-lactobacilli treated and non-infected PAM cells (Mock) were used for comparisons. The expressions of the antiviral factors Mx1, Mx2, OAS1, OAS2 and RNAse L were assessed at 12 h post-infection using RT-qPCR. The results are shown as mean ± SD from three independent biological experiments (n = 3). Significant differences are shown compared to the control group at *p* < 0.05 (*) or *p* < 0.01 (**).

Considering the remarkable immunomodulatory properties of *L. rhamnosus* CRL1505 in the context of respiratory viral infections [5], we hypothesized that the improved resistance of PAM cells against PRRSV infection was related to a differential modulation of antiviral immunity. Then, we evaluated the expressions of IFNs (Figure 3) and antiviral factors (Figure 4) after the viral challenge. PRRSV infection increased the expression of IFN-β and IFN-γ in PAM cells (Figure 3), together with increased expression of Mx1, Mx2, OAS1, OAS2, and RNAse L (Figure 4).

PAM cells pretreated with either live or HK 1505 showed higher expression levels of IFN-β, IFN-γ (Figure 3), Mx1, Mx2, OAS1, OAS2 and RNAse L (Figure 4) than infected control macrophages. In addition, no significant differences in the expression of IFNs and antiviral factors were observed between cells treated with live and heat-killed CRL1505.

**Figure 5:**
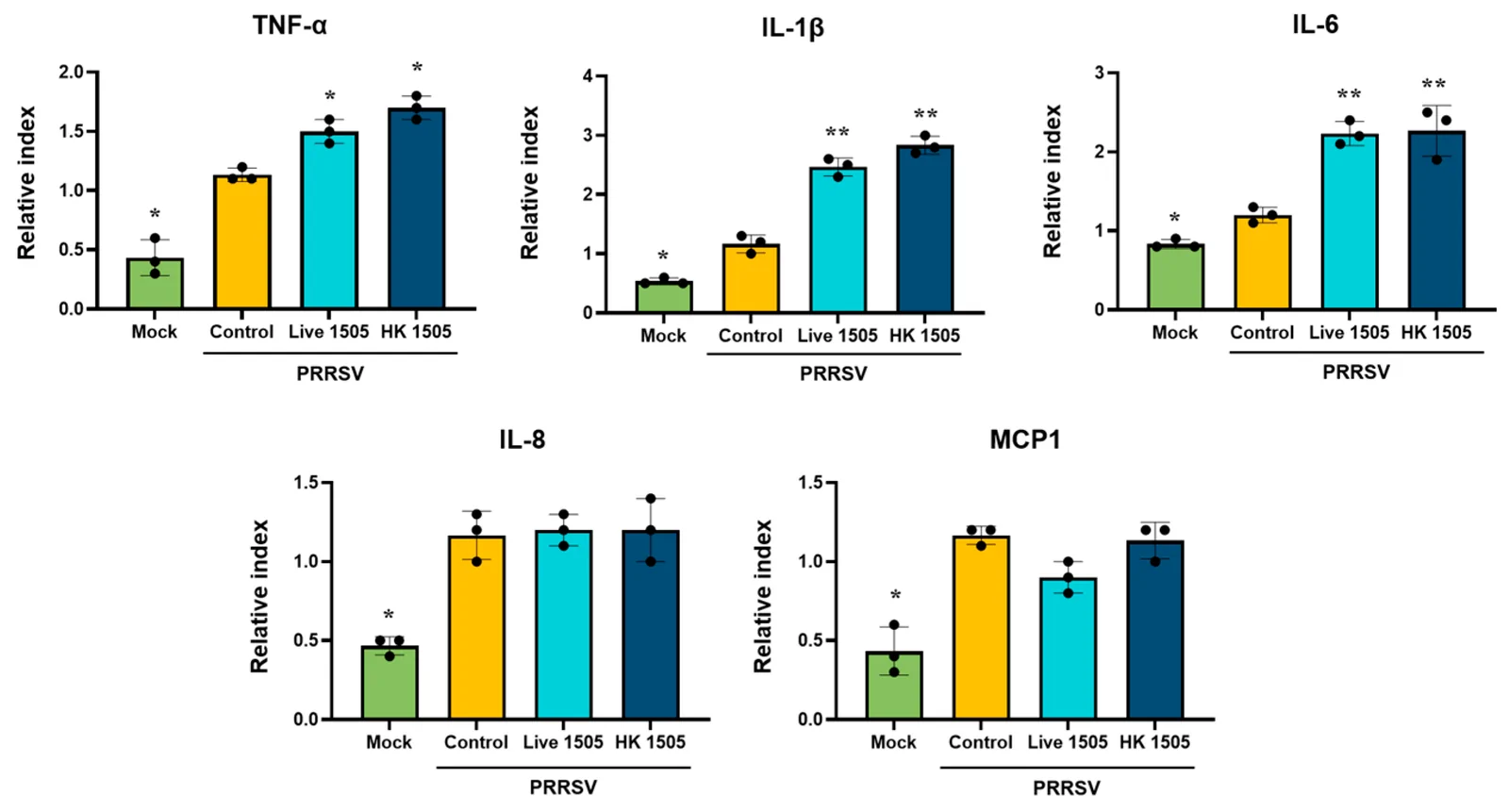
Effect of *Lacticaseibacillus rhamnosus* CRL1505 on the expression of inflammatory cytokines and chemokines in porcine alveolar macrophages after PRRSV infection. Porcine alveolar macrophages (PAM cells) were stimulated with viable (Live 1505) or heat-killed (HK 1505) *L. rhamnosus* CRL1505 for 12 h (MOI = 10) and subsequently infected with PRRSV (MOI = 0.05). PRRSV-infected control cells (Control), and non-lactobacilli treated and non-infected PAM cells (Mock) were used for comparisons. The expressions of TNF-α, IL-1β, IL-6, IL-8, and MCP-1 were assessed at 12 h post-infection using RT-qPCR. The results are shown as mean ± SD from three independent biological experiments (n = 3). Significant differences are shown compared to the control group at *p* < 0.05 (*) or *p* < 0.01 (**).

The changes in the expression of proinflammatory cytokines and chemokines were also evaluated in PRRSV-infected PAM cells (Figure 5). The virus was capable of enhancing the expression of TNF-α, IL-1β, IL-6, IL-8, and MCP-1 in macrophages. It was observed that both live and HK 1505 enhanced TNF-α, IL-1β, and IL-6 compared to control macrophages and with no significant differences between them. Interestingly, neither live nor HK 1505 induced modifications in the expressions of IL-8 and MCP-1 (Figure 5).

**Figure 6:**
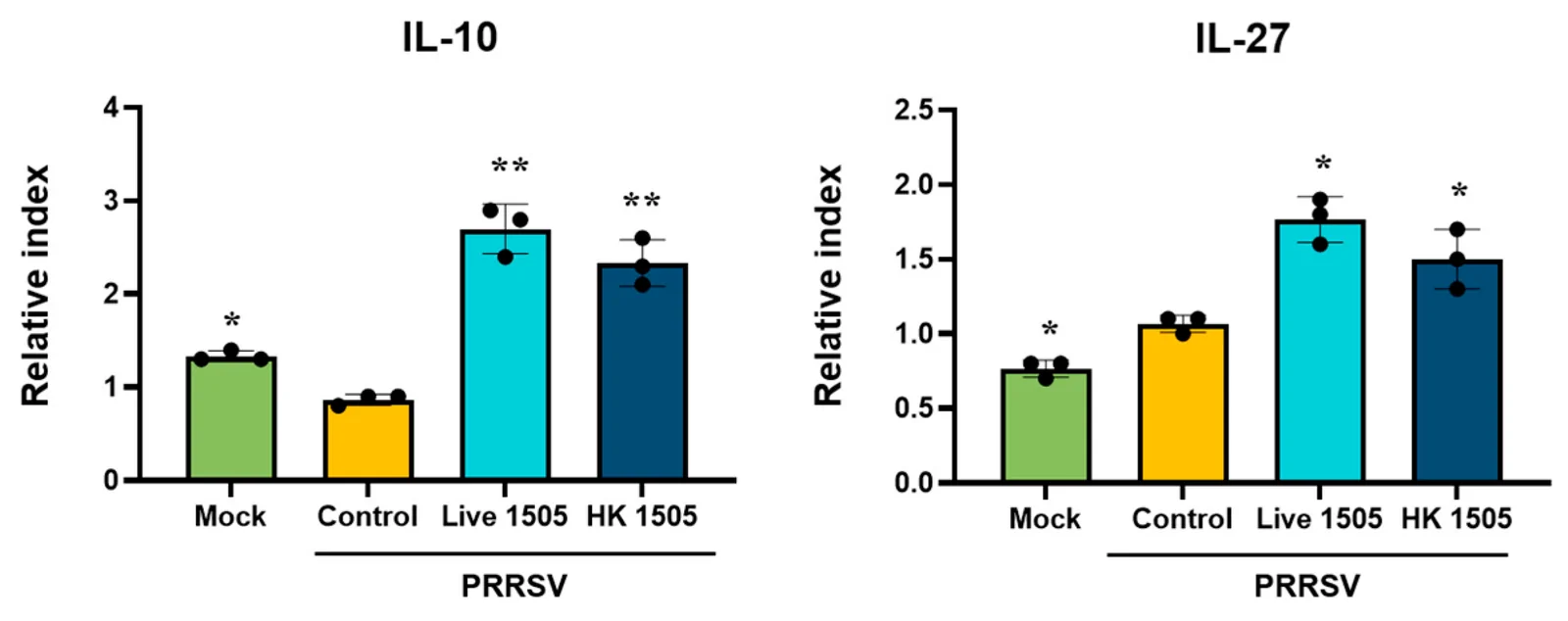
Effect of *Lacticaseibacillus rhamnosus* CRL1505 on the expression of regulatory cytokines in porcine alveolar macrophages after PRRSV infection. Porcine alveolar macrophages (PAM cells) were stimulated with viable (Live 1505) or heat-killed (HK 1505) *L. rhamnosus* CRL1505 for 12 h (MOI = 10) and subsequently infected with PRRSV (MOI = 0.05). PRRSV-infected control cells (Control), and non-lactobacilli treated and non-infected PAM cells (Mock) were used for comparisons. The expressions of IL-10 and IL-27 were assessed at 12 h post-infection using RT-qPCR. The results are shown as mean ± SD from three independent biological experiments (n = 3). Significant differences are shown compared to the control group at *p* < 0.05 (*) or *p* < 0.01 (**).

We next evaluated the expression of the regulatory cytokines IL-10 and IL-27 (Figure 6). PRRSV infection reduced the expression of IL-10, but augmented IL-27 compared with mock cells. Treatment with either live or HK 1505 significantly enhanced IL-10 and IL-27 expression in PRRSV-infected PAM cells. No significant differences were observed between the live and HK 1505 treatments (Figure 6).

**Figure 7:**
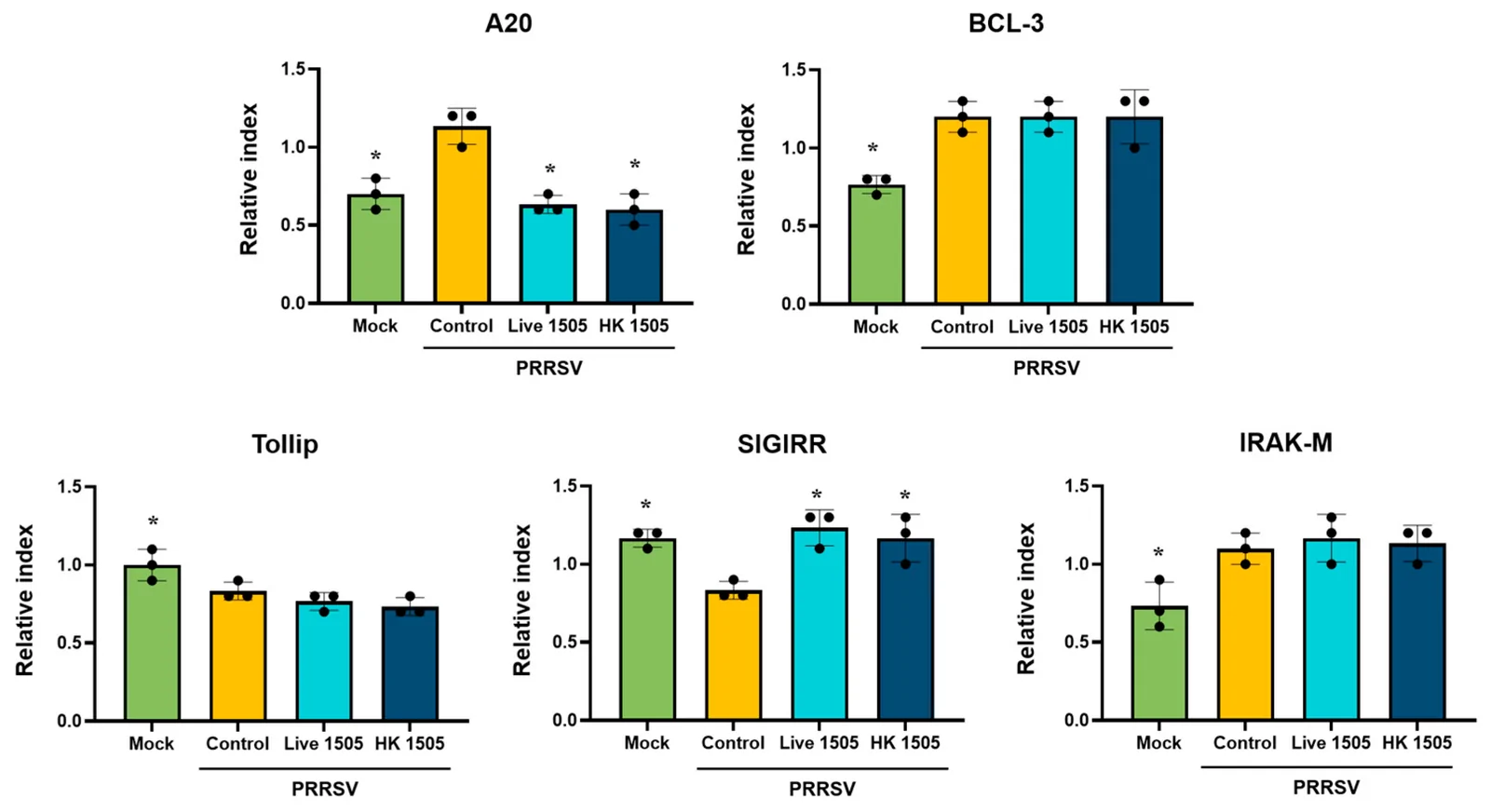
Effect of *Lacticaseibacillus rhamnosus* CRL1505 on the expression of negative regulators of the TLR signaling in porcine alveolar macrophages after PRRSV infection. Porcine alveolar macrophages (PAM cells) were stimulated with viable (Live 1505) or heat-killed (HK 1505) *L. rhamnosus* CRL1505 for 12 h (MOI = 10) and subsequently infected with PRRSV (MOI = 0.05). PRRSV-infected control cells (Control), and non-lactobacilli treated and non-infected PAM cells (Mock) were used for comparisons. The expressions of A20, BCL-3, Tollip, SIGIRR, and IRAK-M were assessed at 12 h post-infection using RT-qPCR. The results are shown as mean ± SD from three independent biological experiments (n = 3). Significant differences are shown compared to the control group at *p* < 0.05 (*).

It was also observed that PRRSV infection reduced the expression of the negative regulators Tollip and SIGIRR in PAM cells, but augmented A20, BCL-3, and IRAK-M compared with mock cells (Figure 7). Treatment with either live or HK 1505 significantly enhanced SIGIRR and reduced A20 expression in PRRSV-infected PAM cells. No significant differences were observed between the live and HK 1505 treatments when the expression levels of SIGIRR and A20 were compared (Figure 7). *L. rhamnosus* CRL1505, neither live nor HK, was able to modulate BCL-3, Tollip or IRAK-M in PRRSV-infected PAM cells.

### 3.4. Lacticaseibacillus rhamnosus CRL1505 Improves Resistance of Porcine Bronchial Epithelial Cells to SIV Infection

**Figure 8:**
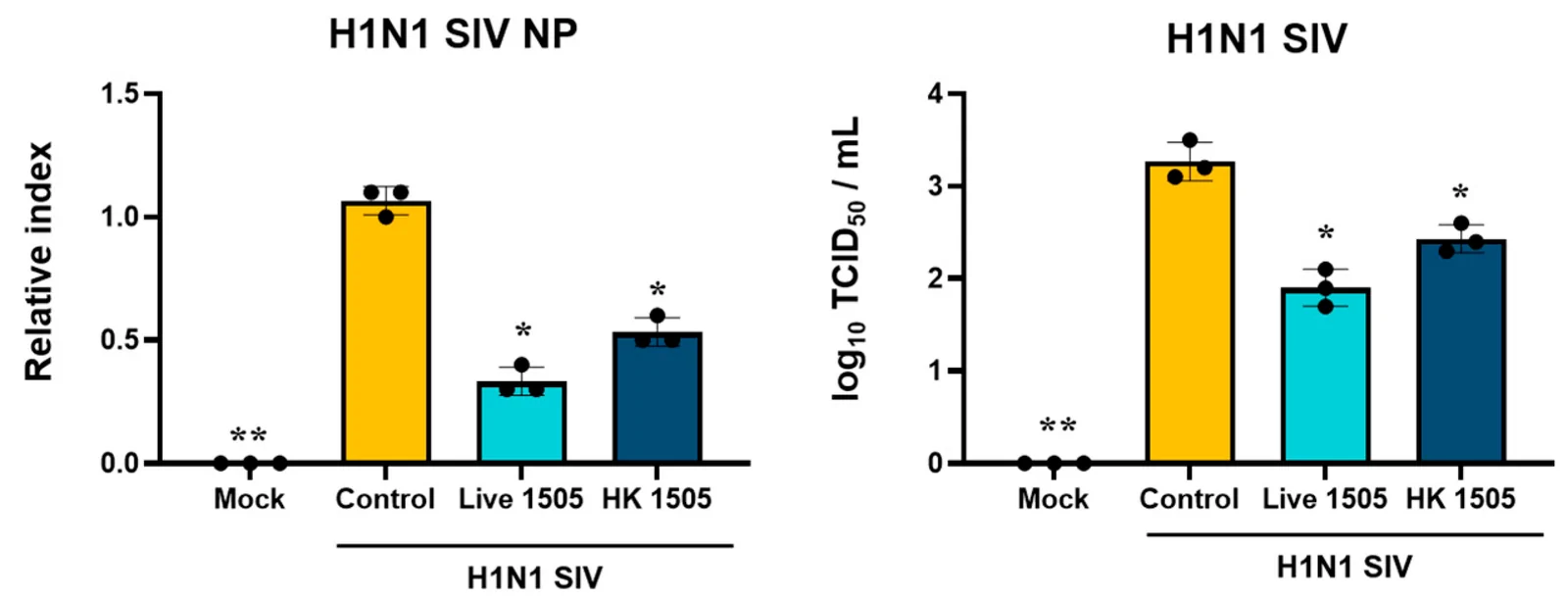
Effect of *Lacticaseibacillus rhamnosus* CRL1505 on SIV H1N1 replication in porcine bronchial epithelial cells. Porcine bronchial epithelial cells (PBE cells) were stimulated with viable (Live 1505) or heat-killed (HK 1505) *L. rhamnosus* CRL1505 for 24 h (MOI = 10) and subsequently infected with SIV H1N1 (MOI = 0.01). SIV-infected control cells (Control), and non-lactobacilli treated and non-infected PBE cells (Mock) were used for comparisons. Viral replication was assessed at 36 h post-infection by measuring the relative expression of SIV H1N1 NP gene using RT-qPCR and by determining SIV titers. The results are shown as mean ± SD from three independent biological experiments (n = 3). Significant differences are shown compared to the control group at *p* < 0.05 (*) or *p* < 0.01 (**).

In a second set of experiments, we evaluated whether the pretreatment of PBE cells with live or HK 1505 could affect their resistance to SIV H1N1 infection (Figure 8). In line with our recent report [14], we demonstrated that PBE cells can be efficiently infected with SIV as confirmed by the detection of the viral NP gene expression and virus titers. Of note, when PBE cells were pretreated with either live or HK 1505, both NP expression and viral titers were significantly lower than infected control PBE cells (Figure 8). Furthermore, no significant differences were found when live- and HK 1505-treated cells were compared.

### 3.5. Lacticaseibacillus rhamnosus CRL1505 Modulates Immune Response to SIV in Porcine Bronchial Epithelial Cells

**Figure 9:**
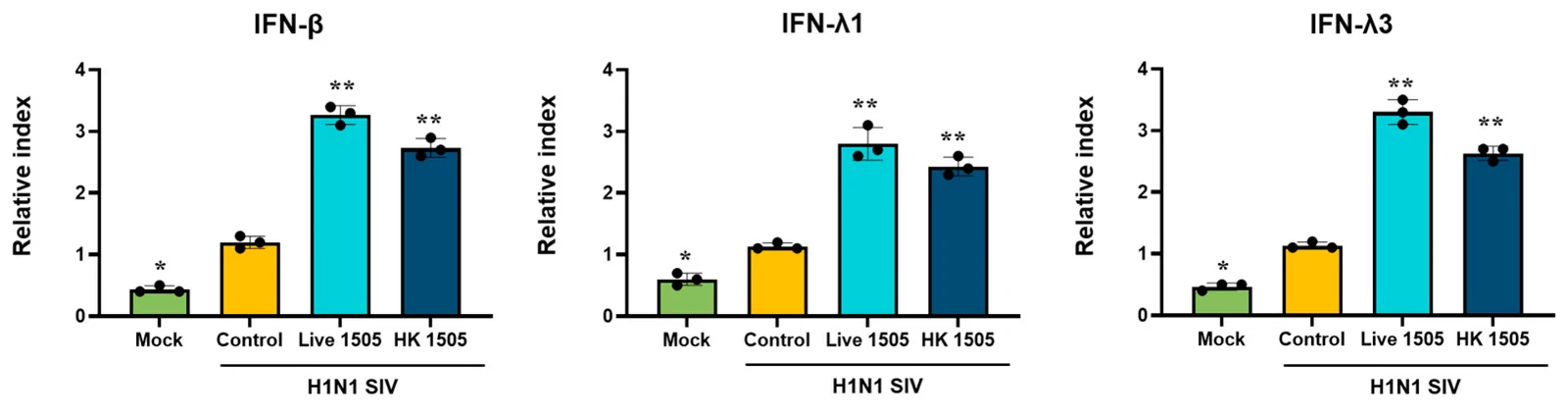
Effect of *Lacticaseibacillus rhamnosus* CRL1505 on the expression of interferons in porcine bronchial epithelial cells after SIV H1N1 infection. Porcine bronchial epithelial cells (PBE cells) were stimulated with viable (Live 1505) or heat-killed (HK 1505) *L. rhamnosus* CRL1505 for 24 h (MOI = 10) and subsequently infected with SIV H1N1 (MOI = 0.01). SIV-infected control cells (Control), and non-lactobacilli treated and non-infected PBE cells (Mock) were used for comparisons. The expressions of IFN-β, IFN-λ1, and IFN-λ3 were assessed at 24 h post-infection using RT-qPCR. The results are shown as mean ± SD from three independent biological experiments (n = 3). Significant differences are shown compared to the control group at *p* < 0.05 (*) or *p* < 0.01 (**).

**Figure 10:**
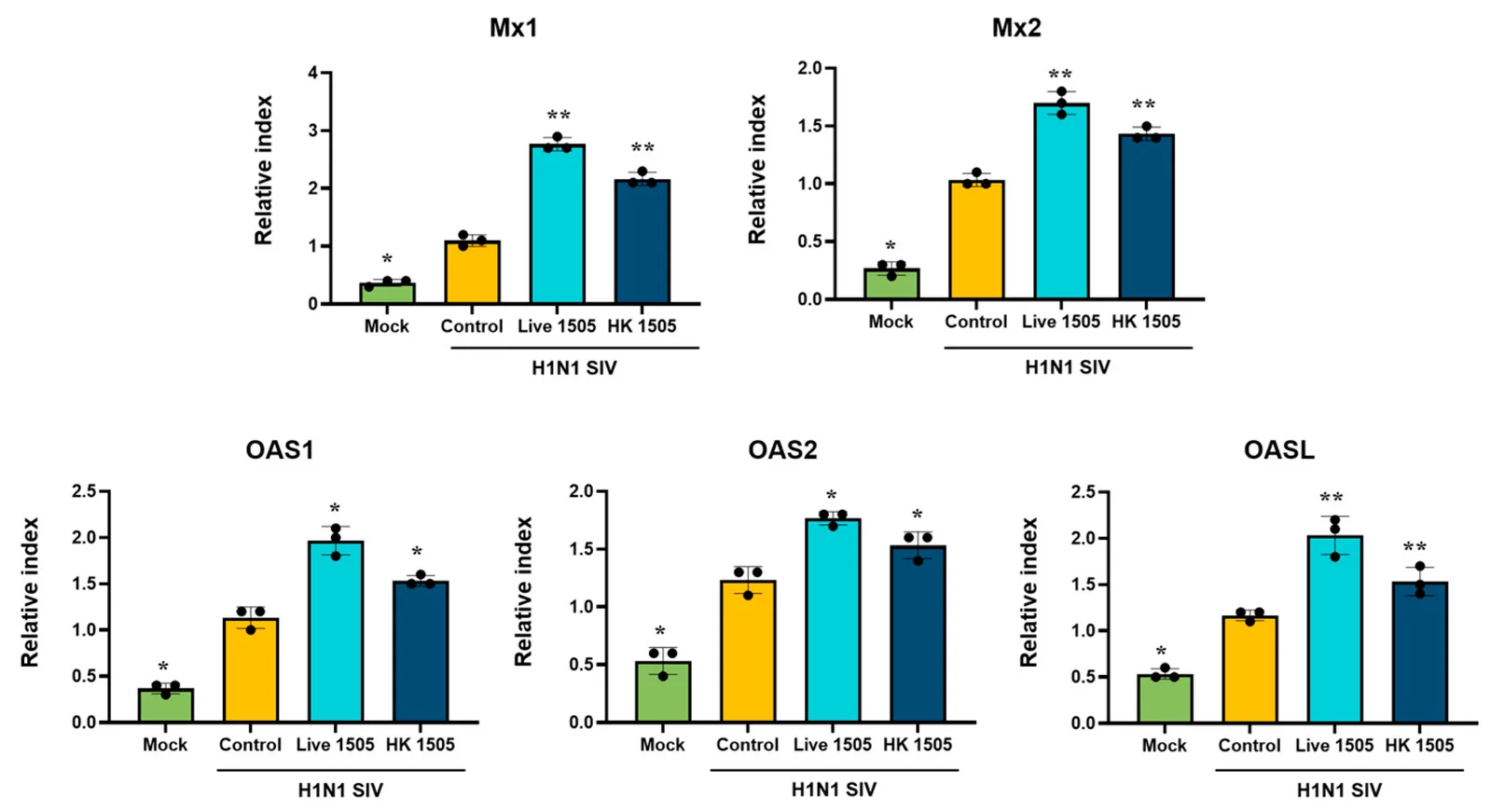
Effect of *Lacticaseibacillus rhamnosus* CRL1505 on the expression of antiviral factors in porcine bronchial epithelial cells after SIV H1N1 infection. Porcine bronchial epithelial cells (PBE cells) were stimulated with viable (Live 1505) or heat-killed (HK 1505) *L. rhamnosus* CRL1505 for 24 h (MOI = 10) and subsequently infected with SIV H1N1 (MOI = 0.01). SIV-infected control cells (Control), and non-lactobacilli treated and non-infected PBE cells (Mock) were used for comparisons. The expressions of Mx1, Mx2, OAS1, OAS2, and OASL were assessed at 24 h post-infection using RT-qPCR. The results are shown as mean ± SD from three independent biological experiments (n = 3). Significant differences are shown compared to the control group at *p* < 0.05 (*) or *p* < 0.01 (**).

We hypothesized that the enhanced resistance of PBE cells to SIV H1N1 infection was associated with the modulation of antiviral immune responses. Therefore, we evaluated the expression of IFNs (Figure 9) and antiviral factors (Figure 10) following viral challenge. SIV infection increased IFN-β, IFN-λ1, and IFN-λ3 expression in PBE cells (Figure 9), accompanied by increased expression of the antiviral factors Mx1, Mx2, OAS1, OAS2, and OASL (Figure 10).

Pretreatment with either live or HK 1505 significantly increased the expression of IFN-β, IFN-λ1, and IFN-λ3 (Figure 9), as well as Mx1, Mx2, OAS1, OAS2, and OASL (Figure 10), compared with infected control PBE cells. No significant differences were observed between live and HK 1505 for any of the evaluated IFNs or antiviral factors.

**Figure 11:**
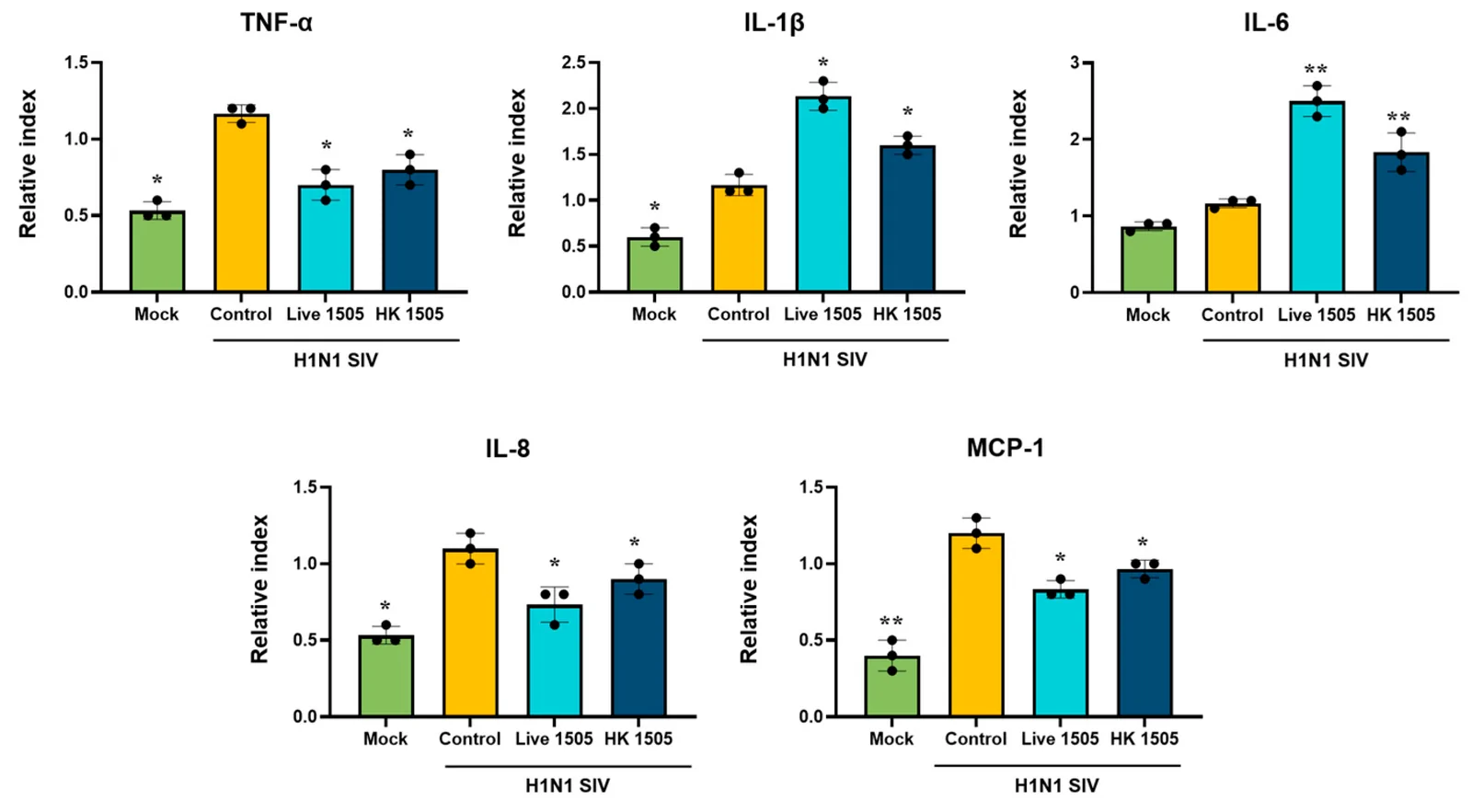
Effect of *Lacticaseibacillus rhamnosus* CRL1505 on the expression of inflammatory cytokines and chemokines in porcine bronchial epithelial cells after SIV H1N1 infection. Porcine bronchial epithelial cells (PBE cells) were stimulated with viable (Live 1505) or heat-killed (HK 1505) *L. rhamnosus* CRL1505 for 24 h (MOI = 10) and subsequently infected with SIV H1N1 (MOI = 0.01). SIV-infected control cells (Control), and non-lactobacilli treated and non-infected PBE cells (Mock) were used for comparisons. The expressions of TNF-α, IL-1β, IL-6, IL-8, and MCP-1 were assessed at 24 h post-infection using RT-qPCR. The results are shown as mean ± SD from three independent biological experiments (n = 3). Significant differences are shown compared to the control group at *p* < 0.05 (*) or *p* < 0.01 (**).

The expression of proinflammatory cytokines and chemokines in SIV-infected PBE cells was next evaluated (Figure 11). SIV H1N1 infection increased the expression of TNF-α, IL-1β, IL-8, and MCP-1 but not IL-6 at the time point evaluated. Pretreatment with either live or HK 1505 significantly reduced TNF-α, IL-8, and MCP-1 expressions compared with infected control cells. In contrast, both treatments improved IL-1β and IL-6 expression compared with infected control PBE cells (Figure 11). Of note, no significant differences between the two treatments were detected.

**Figure 12:**
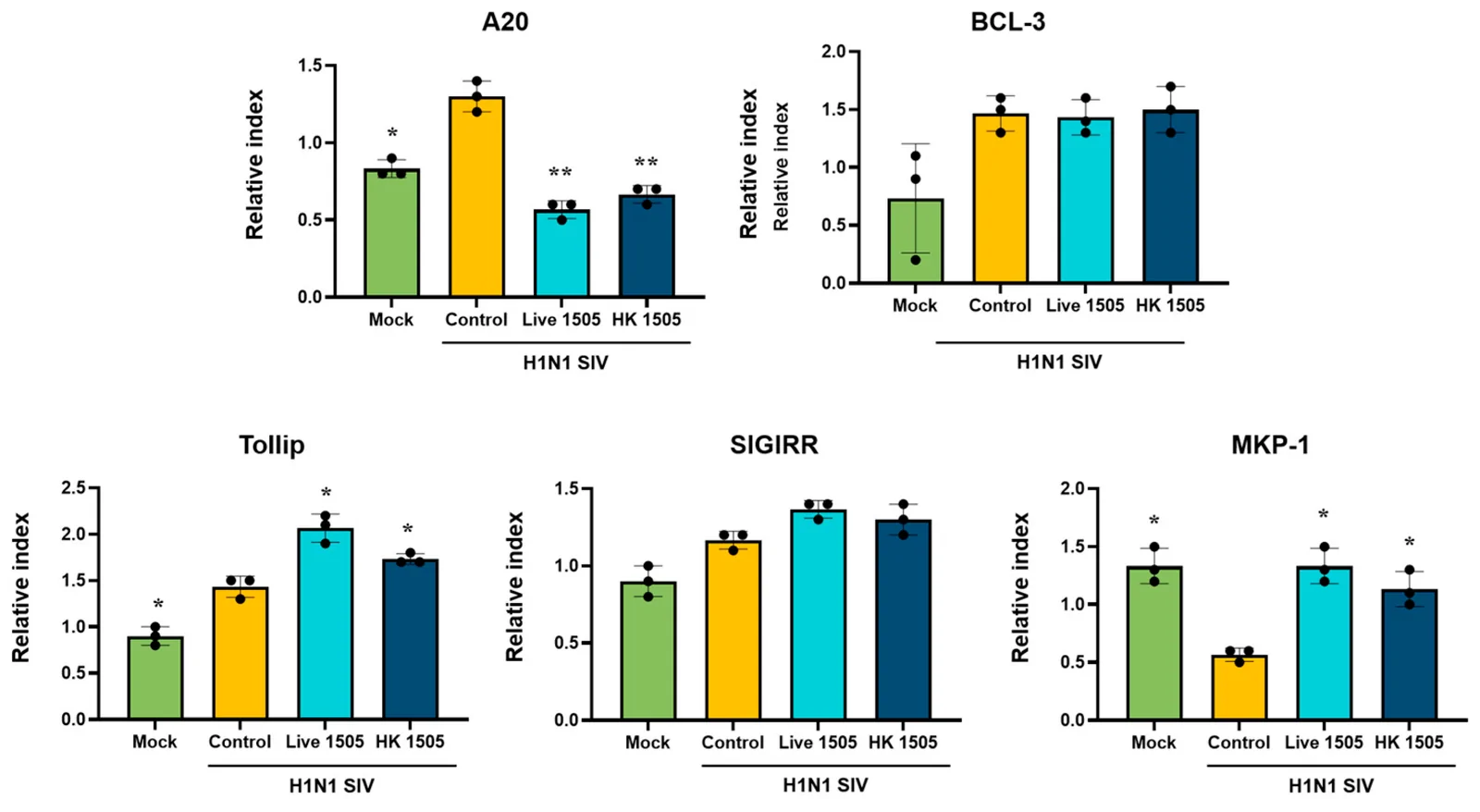
Effect of *Lacticaseibacillus rhamnosus* CRL1505 on the expression of negative regulators of the TLR signaling in porcine bronchial epithelial cells after SIV H1N1 infection. Porcine bronchial epithelial cells (PBE cells) were stimulated with viable (Live 1505) or heat-killed (HK 1505) *L. rhamnosus* CRL1505 for 24 h (MOI = 10) and subsequently infected with SIV H1N1 (MOI = 0.01). SIV-infected control cells (Control), and non-lactobacilli treated and non-infected PBE cells (Mock) were used for comparisons. The expressions of A20, BCL-3, Tollip, SIGIRR, and MKP-1 were assessed at 24 h post-infection using RT-qPCR. The results are shown as mean ± SD from three independent biological experiments (n = 3). Significant differences are shown compared to the control group at *p* < 0.05 (*) or *p* < 0.01 (**).

The expression of the negative regulators A20, BCL-3, Tollip, SIGIRR, and MKP-1 were evaluated in SIV-infected PBE cells (Figure 12). It was also observed that SIV infection increased the expression of A20 and reduced MKP-1 while the remaining negative regulators were not modified. Treatment with either live or HK 1505 significantly enhanced Tollip and MKP-1, while reduced A20 expression compared to control infected PBE cells. No significant differences were observed between the live and HK 1505 treatments when the expression levels of these three negative regulators were compared (Figure 12). *L. rhamnosus* CRL1505, neither live nor HK, was able to modulate BCL-3, or SIGIRR in SIV-infected PBE cells.

### 3.6. CRL1505 Modulates the Kinetics of Interferons and Cytokine Expressions in Virus-Infected Respiratory Cells

Finally, to further characterize the temporal dynamics of the immune responses induced by live and HK 1505, we selected representative immune factors for kinetic analysis at different time points following viral infection. The sampling times were established independently for each experimental model, taking into account the replication kinetics of PRRSV and SIV previously characterized in our studies [12,13].

**Figure 13:**
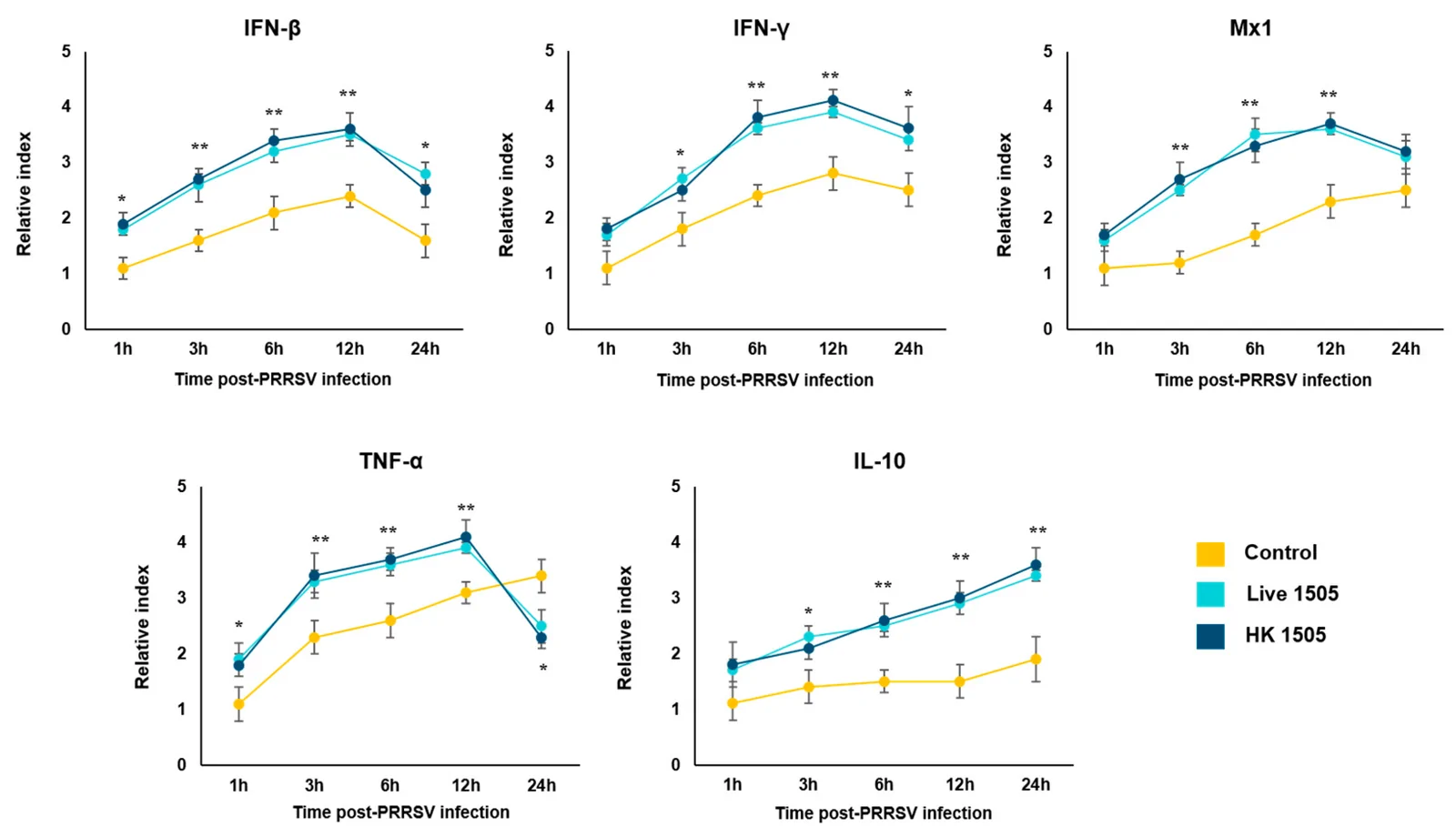
Effect of *Lacticaseibacillus rhamnosus* CRL1505 on the kinetic response of interferons and cytokines in porcine alveolar macrophages after PRRSV infection. Porcine alveolar macrophages (PAM cells) were stimulated with viable (Live 1505) or heat-killed (HK 1505) *L. rhamnosus* CRL1505 for 12 h (MOI = 10) and subsequently infected with PRRSV (MOI = 0.05). PRRSV-infected control cells (Control) were used for comparisons. The expressions of IFN-β, IFN-γ, Mx1, TNF-α, and IL-10 were assessed at 1, 3, 6, 12, and 24 h post-infection using RT-qPCR. The results are shown as mean ± SD from three independent biological experiments (n = 3). Significant differences are shown compared to the control group at *p* < 0.05 (*) or *p* < 0.01 (**).

In PRRSV-PAM cells challenge experiments, both live and HK 1505 enhanced IFN-β expression compared with infected control cells at all the time points evaluated, with the most pronounced differences observed between 6 and 12 h post-infection (Figure 13). IFN-γ expression was significantly increased by the treatments from 3 to 24 h post-infection. Similarly, both treatments enhanced Mx1 expression, with significant differences compared with controls between 3 and 12 h post-infection (Figure 13). Interestingly, the kinetic analysis revealed distinct temporal patterns in the expression of inflammatory and regulatory cytokines. TNF-α expression was significantly higher in live and HK 1505-treated cells during the early stages of infection, reaching its highest levels at 12 h post-infection (Figure 13). However, at 24 h post-infection, TNF-α expression decreased markedly in both treated groups, reaching levels below those observed in infected controls. In contrast, IL-10 expression progressively increased in CRL1505-treated cells, with the greatest differences compared with controls observed at 12 and 24 h post-infection (Figure 13).

**Figure 14:**
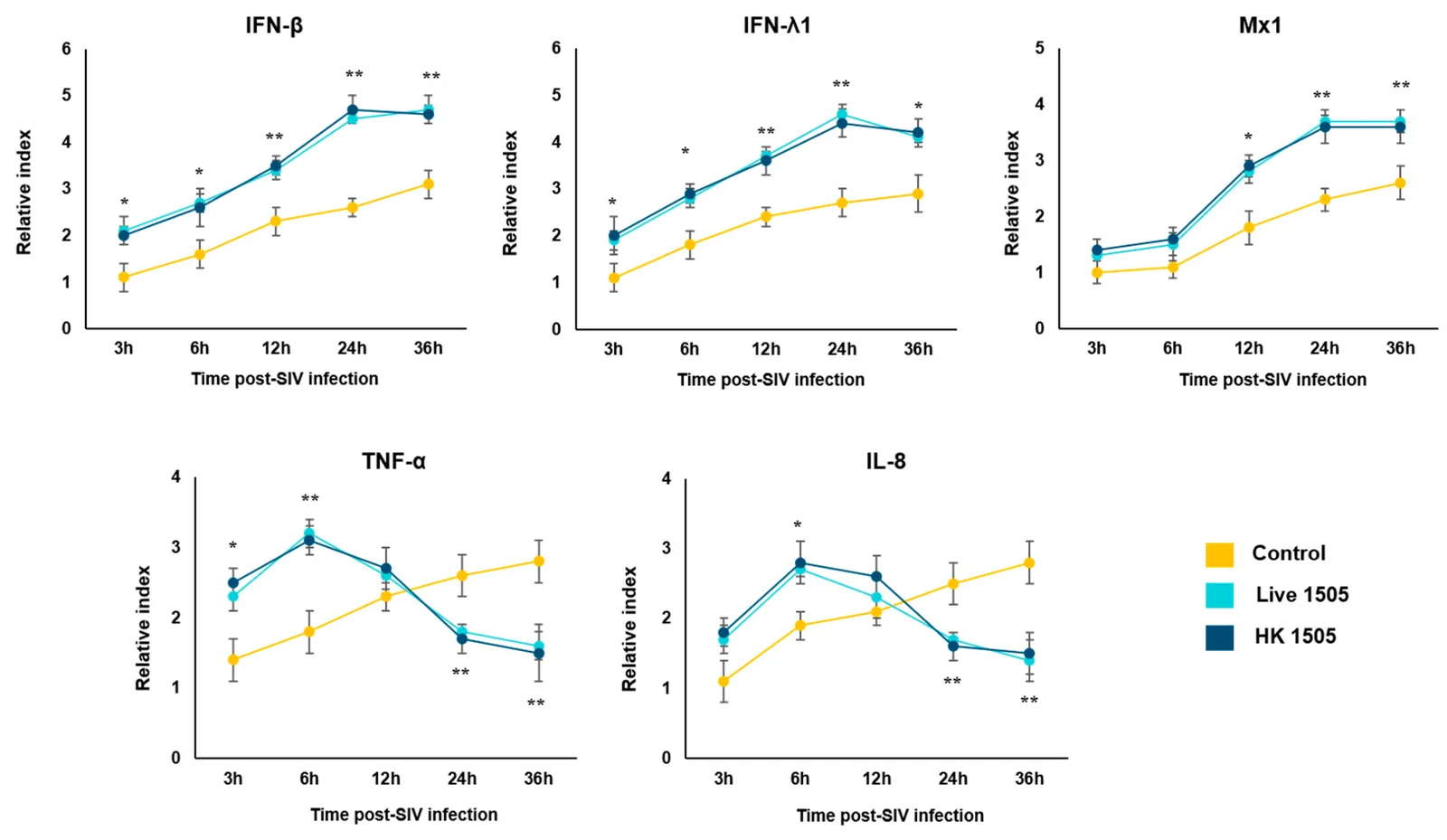
Effect of *Lacticaseibacillus rhamnosus* CRL1505 on the kinetic response of interferons and cytokines in porcine bronchial epithelial cells after SIV H1N1 infection. Porcine bronchial epithelial cells (PBE cells) were stimulated with viable (Live 1505) or heat-killed (HK 1505) *L. rhamnosus* CRL1505 for 24 h (MOI = 10) and subsequently infected with SIV H1N1 (MOI = 0.01). SIV-infected control cells (Control) were used for comparisons. The expressions of IFN-β, IFN-λ1, Mx1, TNF-α, and IL-8 were assessed at 3, 6, 12, 24, and 36 h post-infection using RT-qPCR. The results are shown as mean ± SD from three independent biological experiments (n = 3). Significant differences are shown compared to the control group at *p* < 0.05 (*) or *p* < 0.01 (**).

The kinetic analysis of immune factor expression in SIV-infected PBE cells revealed distinct temporal patterns following pretreatment with live or HK 1505 (Figure 14). Both treatments significantly enhanced IFN-β expression compared with infected controls at all evaluated time points (3–36 h post-infection), with the highest expression levels observed at 24 and 36 h. Similarly, IFN-λ1 expression was significantly increased throughout the experimental period, reaching its maximum at 24 h post-infection. The expression of the antiviral factor Mx1 was also enhanced by both treatments, with significant differences compared with controls from 12 to 36 h post-infection (Figure 14).

Interestingly, the kinetic analysis revealed a time-dependent modulation of inflammatory cytokines and chemokines. TNF-α and IL-8 expressions were significantly higher in CRL1505-treated PBE cells during the early stages of infection, particularly at 3 and 6 h post-infection for TNF-α and at 6 h for IL-8 (Figure 14). However, this pattern was reversed at later time points, with TNF-α and IL-8 expressions decreasing below control levels at 24 and 36 h post-infection. No differences were observed between live and HK 1505 for the evaluated immune factors.

## 4. Discussion

Despite this accumulated evidence of the beneficial immunomodulatory effects of *L. rhamnosus* CRL1505 in the respiratory tract [5], the direct effects of this strain on distinct cellular components of the respiratory innate immune system remain incompletely characterized. The present study demonstrates that the CRL1505 strain enhances resistance to viral infection in two distinct respiratory cell populations: RECs and alveolar macrophages. Both viable and HK 1505 reduced PRRSV infection in PAM cells and SIV replication in PBE cells and enhanced the expression of IFNs and antiviral factors. In addition, the study of the inflammatory and regulatory cytokine responses indicated that CRL1505 does not induce a uniform pro- or anti-inflammatory response but rather modulates the kinetics of cytokine’s expressions.

**Figure 15:**
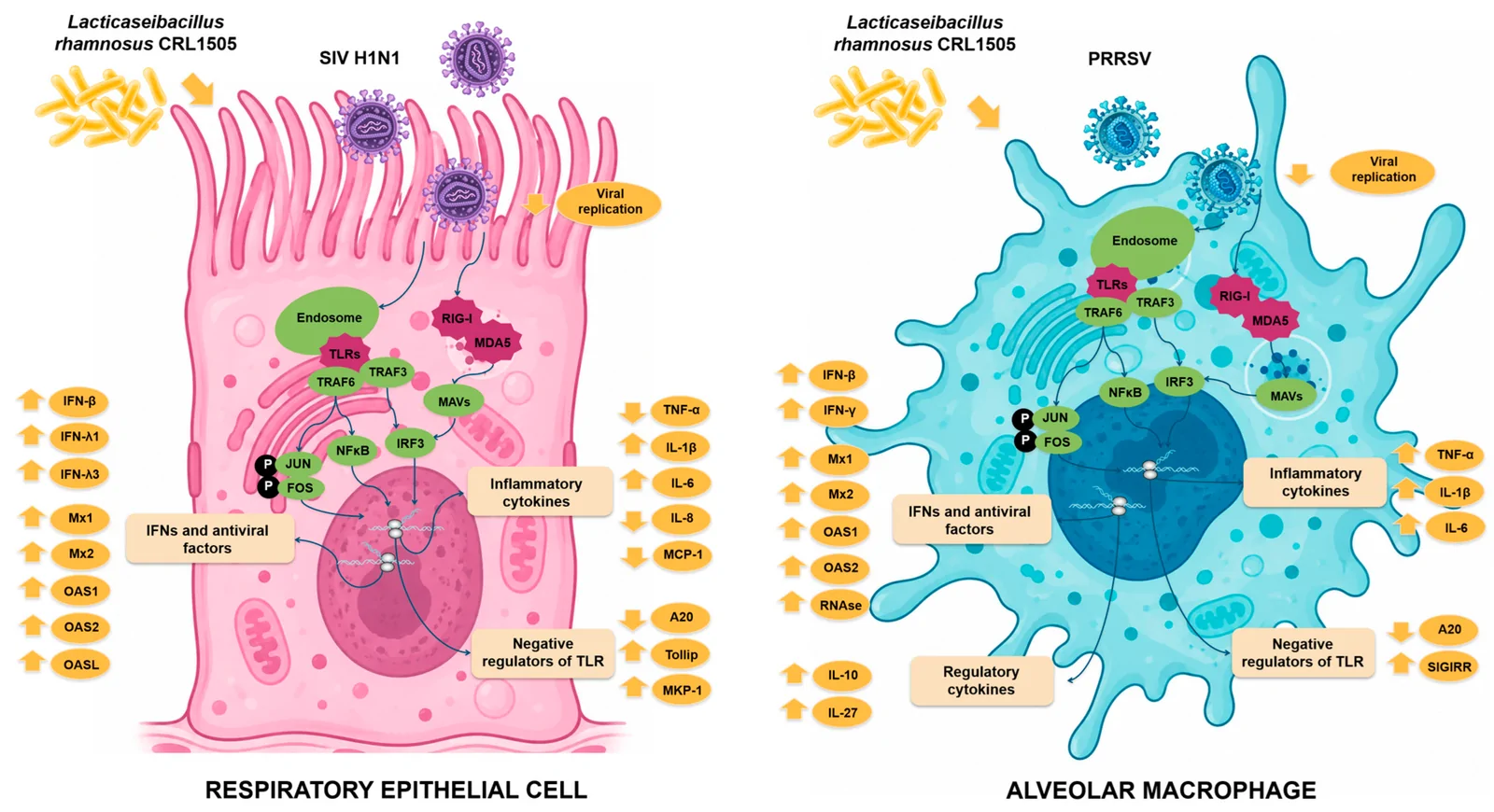
Schematic representation of the immunomodulatory effects of *Lacticaseibacillus rhamnosus* CRL1505 on porcine bronchial epithelial cells (PBE cells) challenged with swine influenza virus (SIV H1N1) and porcine alveolar macrophages (PAM cells) infected with porcine reproductive and respiratory syndrome virus (PRRSV). In PBE cells (**left** panel), pretreatment with viable or heat-killed CRL1505 enhanced the expression of IFN-β, IFN-λ1, and IFN-λ3, together with the antiviral factors Mx1, Mx2, OAS1, OAS2, and OASL. These responses were accompanied by increased IL-1β and IL-6 expression, reduced TNF-α, IL-8, and MCP-1 expression, increased Tollip and MKP-1 expression, and decreased A20 expression. In PAM cells (**right** panel), CRL1505 enhanced IFN-β and IFN-γ expression and increased the expression of Mx1, Mx2, OAS1, OAS2, and RNase L. These changes were accompanied by increased expression of the inflammatory cytokines TNF-α, IL-1β, and IL-6, the regulatory cytokines IL-10 and IL-27, and the negative regulator SIGIRR, together with decreased A20 expression. The schematic illustrates the cell type-specific modulation of antiviral, inflammatory, and regulatory responses induced by CRL1505. Arrows indicate the direction of gene expression changes relative to virus-infected control cells. Intracellular signaling components are shown schematically to illustrate potential regulatory pathways and do not represent direct measurements of their activation.

Consistent with our previous studies [7,8,11], in which nasal administration of *L. rhamnosus* CRL1505 enhanced IFN-β and IFN-γ production in the respiratory tract together with increased expression of antiviral factors, the present results showed that both live and HK 1505 strengthened IFNs and antiviral responses in the two respiratory cell models (Figure 15).

The enhancement of antiviral defenses induced by CRL1505 is consistent with our previous in vivo findings in mice infected with RSV. In those studies, alveolar macrophages from mice nasally treated with *L. rhamnosus* CRL1505 exhibited increased expression of IFNs and antiviral factors, including OAS1, Mx2, RNAse L, and IFITM3, compared with untreated controls [7,11]. The timely induction of type I IFNs, IFN-stimulated genes, and IFN-γ in alveolar macrophages plays an important role in antiviral protection [17,18]. These responses activate complementary antiviral mechanisms, including the OAS–RNAse L pathway, which promotes viral RNA degradation, and IFITM3, which interferes with early stages of the viral life cycle [18]. Increased Mx2 expression in the respiratory tract has also been associated with enhanced resistance to RSV. A similar relationship between IFNs responses and viral control is relevant to PRRSV infection [19,20,21]. These responses promote antiviral effectors such as Mx1 and OAS1, which have been reported to restrict PRRSV replication and cytopathic effects [22,23]. An important aspect of the antiviral activity of CRL1505 is its capacity to stimulate IFN responses before viral infection. The analysis performed in the present study showed that CRL1505 enhanced IFN expression in PAM cells prior to PRRSV challenge. This observation is particularly relevant considering that PRRSV employs multiple mechanisms to suppress type I IFN production and signaling [20,21]. The early induction of IFNs by CRL1505 may therefore contribute to establishing an antiviral state before the virus can effectively interfere with host innate immune responses. This mechanism could, at least in part, explain the enhanced expression of antiviral factors and the reduced PRRSV-associated molecular and antigenic markers observed in CRL1505-treated PAM cells. Nevertheless, additional mechanistic studies will be required to determine whether CRL1505 directly counteracts specific PRRSV immune evasion mechanisms or primarily acts through the establishment of a pre-existing antiviral state.

Thus, the increased expression of IFN-β, IFN-γ, and multiple antiviral factors observed in CRL1505-treated PAM cells is consistent with the reduced PRRSV ORF5 expression and viral antigen detection observed in the present study. However, although the reduction in these parameters supports an antiviral effect of CRL1505 in PAM cells, infectious viral titers were not determined in this model. Therefore, additional studies assessing infectious PRRSV production will be necessary to establish whether these changes translate into reduced viral infectivity.

The antiviral activity of *L. rhamnosus* CRL1505 was not restricted to alveolar macrophages, as both live and HK 1505 also enhanced antiviral responses and reduced SIV replication in PBE cells. This finding is consistent with previous studies showing that immunobiotics can modulate innate antiviral responses in RECs [24,25]. *L. rhamnosus* D3189, for example, reduced RSV shedding in primary human nasal epithelial cells and enhanced IFN-β production following viral challenge [25]. Similarly, we previously demonstrated that *L. plantarum* MPL16 and CRL1506 modulated IFN-β and antiviral factor expression in Calu-3 cells (human RECs) challenged with poly(I:C) or SARS-CoV-2 [24]. In the present study, pretreatment with either live or HK 1505 enhanced IFN-β, IFN-λ1, and IFN-λ3 before and after SIV challenge. Type I and type III IFNs are important components of epithelial antiviral immunity and promote the expression of IFN-stimulated genes that restrict influenza virus replication. Among these effectors, Mx proteins interfere with influenza virus replication, whereas the OAS pathway contributes to viral RNA degradation through activation of RNAse L [26,27,28]. Thus, the enhanced IFNs and antiviral factor response induced by CRL1505 in PBE cells was associated with the reduced SIV replication observed in the present study.

In addition to enhancing antiviral defenses, *L. rhamnosus* CRL1505 differentially modulated inflammatory and regulatory responses in PAM and PBE cells. During respiratory viral infections, alveolar macrophages must mount effective antiviral responses while tightly regulating inflammation to promote viral clearance without causing excessive tissue injury. In previous studies, we demonstrated that nasal administration of immunobiotic lactobacilli enhanced the capacity of murine alveolar macrophages to produce IL-6 and IL-27 following TLR3 activation. These cytokines contributed to the induction of IL-10-producing regulatory T cells and were associated with improved control of pulmonary inflammation and reduced tissue injury [7,11]. Thus, the beneficial effects of immunobiotics in the respiratory tract appear to involve not only enhancement of antiviral defenses but also regulation of the balance between inflammatory and regulatory responses.

In this work, PRRSV infection induced a marked inflammatory response in PAM cells, increasing TNF-α, IL-1β, IL-6, IL-8, and MCP-1 expression. This is consistent with previous studies linking PRRSV infection, particularly infection with highly pathogenic variants, with increased production of inflammatory mediators such as IL-1β and IL-6 and more severe pulmonary inflammation [29,30]. At 12 h post-infection, pretreatment with live or HK CRL1505 further enhanced TNF-α, IL-1β, and IL-6 expression, accompanied by increased expression of the regulatory cytokines IL-10 and IL-27. Importantly, the additional kinetic analyses revealed a time-dependent modulation of TNF-α and IL-10 expression. During the early stages, CRL1505-treated PAM cells exhibited higher TNF-α expression than controls, whereas IL-10 expression remained comparable between groups. At later time points, however, TNF-α expression was reduced in CRL1505-treated cells relative to controls, while IL-10 expression was markedly enhanced. These findings suggest that CRL1505 modulates not only the magnitude but also the temporal dynamics of inflammatory and regulatory cytokine responses.

A distinct transcriptional profile was also induced by *L. rhamnosus* CRL1505 in PBE cells. During influenza infection, epithelial cells contribute to host defense by producing inflammatory cytokines and chemokines that recruit and activate immune cells; however, excessive production of these mediators can also contribute to disruption of the epithelial–endothelial barrier and pulmonary tissue injury [31,32,33]. In SIV-infected PBE cells, both live and HK CRL1505 reduced TNF-α, IL-8, and MCP-1 expression while increasing IL-1β and IL-6. Additional kinetic analyses revealed a time-dependent modulation of inflammatory mediators, with TNF-α and IL-8 expression showing higher levels in CRL1505-treated cells at early time points, followed by lower levels compared with controls at later stages. These findings suggest that CRL1505 modulates not only the expression of individual cytokines and chemokines in RECs but also their temporal dynamics, potentially favoring an early inflammatory response followed by its attenuation.

Taken together, these findings indicate that *L. rhamnosus* CRL1505 does not exert a uniform pro- or anti-inflammatory effect across the two respiratory infection models but instead induces distinct, time-dependent immunomodulatory responses. These temporal patterns of differential modulation of inflammatory and regulatory mediators suggest that CRL1505 may promote an initial inflammatory activation followed by a shift toward a more regulated transcriptional profile. This interpretation is consistent with our previous in vivo findings demonstrating the beneficial immunomodulatory effects of CRL1505 in the respiratory tract [7,8,9,10,11]. Nevertheless, the functional contribution of these transcriptional changes to antiviral protection and the control of inflammatory tissue damage remains to be established.

The differential modulation of inflammatory responses by CRL1505 was also reflected in the expression of negative regulators of innate immune signaling. These molecules play an important role in limiting excessive inflammatory responses while preserving effective host defense [34]. Among them, A20 (TNFAIP3) is a ubiquitin-editing protein that negatively regulates several innate immune signaling pathways, including TLR3- and RIG-I-mediated activation of IRF3. Previous studies have shown that influenza infection induces A20 expression in RECs and in the lung [35,36,37], suggesting that its upregulation may represent a mechanism for controlling virus-induced innate immune activation. In the present study, SIV infection increased A20 expression in PBE cells, whereas pretreatment with either live or HK 1505 reduced its expression. Interestingly, CRL1505 also reduced A20 expression in PRRSV-infected PAM cells. Considering the inhibitory effect of A20 on IRF3 signaling, its reduced expression may contribute to the enhanced IFN and antiviral factor responses observed in both respiratory cell populations. This interpretation is consistent with our previous observations in intestinal epithelial cells, where changes in A20 expression were associated with the modulation of IRF3 signaling [38]. However, the present findings demonstrate an association between reduced A20 expression and enhanced antiviral responses, without establishing a causal relationship. Direct assessment of IRF3 activation and functional manipulation of A20 will be necessary to determine whether this regulatory pathway contributes to CRL1505-mediated antiviral effects in respiratory cells.

In contrast to the common reduction in A20, CRL1505 differentially modulated the expression of other negative regulators according to the respiratory cell type. In PBE cells, both live and HK 1505 increased Tollip and MKP-1 expression. Tollip has been implicated in the negative regulation of TLR- and IL-1-mediated signaling and the control of virus-induced pulmonary inflammation [39,40], whereas MKP-1 regulates inflammatory signaling through MAPK dephosphorylation [36]. In PAM cells, CRL1505 increased SIGIRR expression, which has an established inhibitory role in TLR signaling [41]. Taken together, these findings allow us to speculate that the distinct transcriptional profiles of innate immune regulators in CRL1505-treated RECs and alveolar macrophages may be associated with the expressions of antiviral factors and cytokines in a quantitative and/or kinetic way. Although this speculation is consistent with the known immunoregulatory functions of A20, Tollip, MKP-1, and SIGIRR, their functional involvement in the observed antiviral and inflammatory responses remains hypothetical. The present study did not assess the protein abundance, activation, or functional contribution of these regulators. Therefore, additional experiments involving protein-level measurements and targeted functional approaches will be required to establish whether these molecules mediate CRL1505-induced immunomodulation.

An important finding of the present study was that HK 1505 retained the capacity to enhance antiviral responses in both RECs and alveolar macrophages. Both viable and heat-killed preparations exerted significant effects compared with infected controls, and their immunomodulatory profiles were generally similar for most of the evaluated parameters. Although these observations do not establish formal biological equivalence, they indicate that bacterial viability is not strictly required for the antiviral and immunomodulatory activities of *L. rhamnosus* CRL1505. This observation is consistent with previous studies showing that viable and HK 1505 can exert similar immunomodulatory and protective effects in the respiratory tract, including enhanced antiviral immunity and resistance to RSV infection [7]. However, the relative activity of viable and heat-killed bacteria cannot be generalized across immunobiotic strains or biological responses. For example, heat-killed *Limosilactobacillus reuteri* PSC102 induced stronger phagocytic and inflammatory responses in macrophages than its viable counterpart [42], whereas viable *Lactiplantibacillus plantarum* CRL1506 provided greater protection against TLR3-mediated lung injury than the heat-killed preparation, despite both forms improving resistance to RSV infection [7]. Similarly, heat-killed *Lacticaseibacillus paracasei* 6235 showed greater activity than viable cells for selected outcomes in an experimental model of intestinal inflammation [43]. Thus, bacterial inactivation should not be assumed to simply preserve or attenuate the biological activity of an immunobiotic strain. Rather, heat treatment may maintain, reduce, or even enhance specific effects depending on the bacterial strain, cellular target, and biological outcome evaluated. These observations highlight the importance of directly comparing viable and non-viable preparations under the same experimental conditions when characterizing the immunomodulatory potential of candidate immunobiotics.

Although the present findings complement previous in vivo studies demonstrating the protective effects of CRL1505, immortalized cell lines cannot fully reproduce the complexity of the respiratory environment, particularly the interactions between epithelial and immune cells. Future studies using primary respiratory cells, organoids, or epithelial–immune cell co-culture systems could provide additional insights into these interactions and further clarify the cellular mechanisms underlying CRL1505-mediated respiratory protection. Another limitation of the present study is that the expression of immune factors was evaluated at the transcriptional level. Although these analyses revealed consistent immunomodulatory patterns associated with reduced viral markers or infectious viral titers, changes in mRNA expression do not necessarily correspond to changes in protein abundance or secretion. Therefore, future studies incorporating protein-level analyses will be valuable to further characterize the antiviral mechanisms induced by CRL1505 in respiratory cells. An additional limitation is that different viruses and experimental conditions were used for PAM and PBE cells. Consequently, the differences in immune responses observed between these models cannot be attributed exclusively to intrinsic cellular characteristics, as virus-specific effects and experimental conditions may also contribute. The present study was designed to characterize CRL1505-mediated immunomodulation within two complementary respiratory infection models rather than to establish direct quantitative differences between the two cell types.

## 5. Conclusions

In conclusion, *Lacticaseibacillus rhamnosus* CRL1505 enhanced resistance to viral infection in both RECs and alveolar macrophages. Viable and HK 1505 increased IFNs and antiviral factor expression and reduced viral infection in PBE and PAM cells, while inducing distinct inflammatory and regulatory profiles in the two respiratory infection models. These findings suggest that CRL1505 promotes an improved antiviral response accompanied by modulation of inflammation. Importantly, HK 1505 retained the antiviral and immunomodulatory activities observed with the viable strain, supporting the further investigation of non-viable CRL1505 preparations for respiratory immunomodulation.

The novelty of the present study lies in advancing our understanding of the cellular responses underlying the respiratory immunomodulatory activity of *L. rhamnosus* CRL1505 previously demonstrated in vivo. By examining two complementary respiratory infection models, we provide evidence that CRL1505 directly modulates antiviral responses in both RECs and alveolar macrophages, with distinct immunomodulatory profiles depending on the experimental context. Importantly, our findings demonstrate that bacterial viability is not essential for the antiviral and immunomodulatory activities observed under the conditions evaluated. Furthermore, the kinetic analyses provide new insights into the early induction of antiviral defenses and the temporal modulation of inflammatory and regulatory mediators following viral challenge. Together, these findings extend previous in vivo observations by identifying cellular responses potentially contributing to CRL1505-mediated respiratory protection, while providing a rationale for further mechanistic investigation of non-viable CRL1505 preparations.

## Data Availability

The data presented in this study are available throughout the article.

## Supplementary Materials

The following supporting information can be downloaded at: https://www.mdpi.com/article/10.3390/biology15211927/s1; Supplementary Figure S1. Effect of *Lacticaseibacillus rhamnosus* CRL1505 on the expression of interferons and cytokines in porcine alveolar macrophages. Porcine alveolar macrophages (PAM cells) were stimulated with viable (Live 1505) or heat-killed (HK 1505) *L. rhamnosus* CRL1505 for 12 h (MOI = 10). Non-lactobacilli treated PAM cells (Mock) were used for comparisons. The expressions of interferons, proinflammatory cytokines and chemokines and regulatory cytokines were assessed using RT-qPCR. The results are shown as mean ± SD from three independent biological experiments (n = 3). Significant differences are shown compared to the control group at *p* < 0.05 (*) or *p* < 0.01 (**). Supplementary Figure S2. Effect of *Lacticaseibacillus rhamnosus* CRL1505 on the expression of interferons and cytokines in porcine respiratory epithelial cells. Porcine bronchial epithelial cells (PBE cells) were stimulated with viable (Live 1505) or heat-killed (HK 1505) *L. rhamnosus* CRL1505 for 24 h (MOI = 10). Non-lactobacilli treated PBE cells (Mock) were used for comparisons. The expressions of interferons, proinflammatory cytokines and chemokines and regulatory cytokines were assessed using RT-qPCR. The results are shown as mean ± SD from three independent biological experiments (n = 3). Significant differences are shown compared to the control group at *p* < 0.05 (*) or *p* < 0.01 (**).

## Abbreviations

The following abbreviations are used in this manuscript:

A20 (TNFAIP3)	TNF alpha-induced protein 3
BCL-3	B-cell lymphoma 3
DMEM	Dulbecco’s Modified Eagle Medium
FCS	Fetal calf serum
HK	Heat-killed
IAV	Influenza A virus
IFA	Immunofluorescence assay
IFITM3	Interferon-induced transmembrane protein 3
IFN	Interferon
IL	Interleukin
IRAK-M	IL-1 receptor-associated kinase M
MCP-1	Monocyte chemoattractant protein-1
MDCK	Madin–Darby canine kidney (cells)
MKP-1	MAP kinase phosphatase-1
MOI	Multiplicity of infection
MRS	de Man, Rogosa and Sharpe (agar)
Mx1, Mx2	Myxovirus resistance protein 1, 2
NP	Nucleoprotein
OAS1, OAS2	2′,5′-oligoadenylate synthetase 1, 2
OASL	2′-5′-oligoadenylate synthetase like
ORF5	Open reading frame 5
PAM	Porcine alveolar macrophage(s)
PBE	Porcine bronchial epithelial (cells)
PBS	Phosphate-buffered saline
PRRSV	Porcine reproductive and respiratory syndrome virus
REC	Respiratory epithelial cell(s)
RNase L	Ribonuclease L
RPMI	Roswell Park Memorial Institute (medium)
RSV	Respiratory syncytial virus
RT-qPCR	Reverse transcription quantitative polymerase chain reaction
SEM	Scanning electron microscopy
SIGIRR	Single immunoglobulin interleukin-1 receptor-related molecule
SIV	Swine influenza virus
SPF	Specific pathogen-free
TCID_50_	50% tissue culture infectious dose
TLR	Toll-like receptor
TNF-α	Tumor necrosis factor alpha
Tollip	Toll-interacting protein
TPCK	Tosylsulfonyl phenylalanyl chloromethyl ketone
