# Supplementary material for: Lacticaseibacillus rhamnosus CRL1505 Enhances Antiviral State in Respiratory Epithelial Cells and Alveolar Macrophages
Source: Biology (Basel). 2026 Oct 23;15(21):1927. doi: 10.3390/biology15211927 (PMC13762214; doi:10.3390/biology15211927)
Supplement: Supplementary file 1 [file biology-15-01927-s001.zip › biology-4609718-supplementary.pdf]

*Article*

# *Lacticaseibacillus rhamnosus* CRL1505 Enhances Antiviral State in Respiratory Epithelial Cells and Alveolar Macrophages

**Luciano Arellano-Arriagada [1,2]†, Ayelen Antonella Baillo [3]†, Leonardo Albarracin[1], Solange Cisterna-Vergara [1,2], Xi-Chen Bai [1,2], Wenxiao Du [1,2], Weichen Gong [2,4], Yoshihito Suda[5], Haruki Kitazawa[2,4,]* and Julio Villena[1,]***

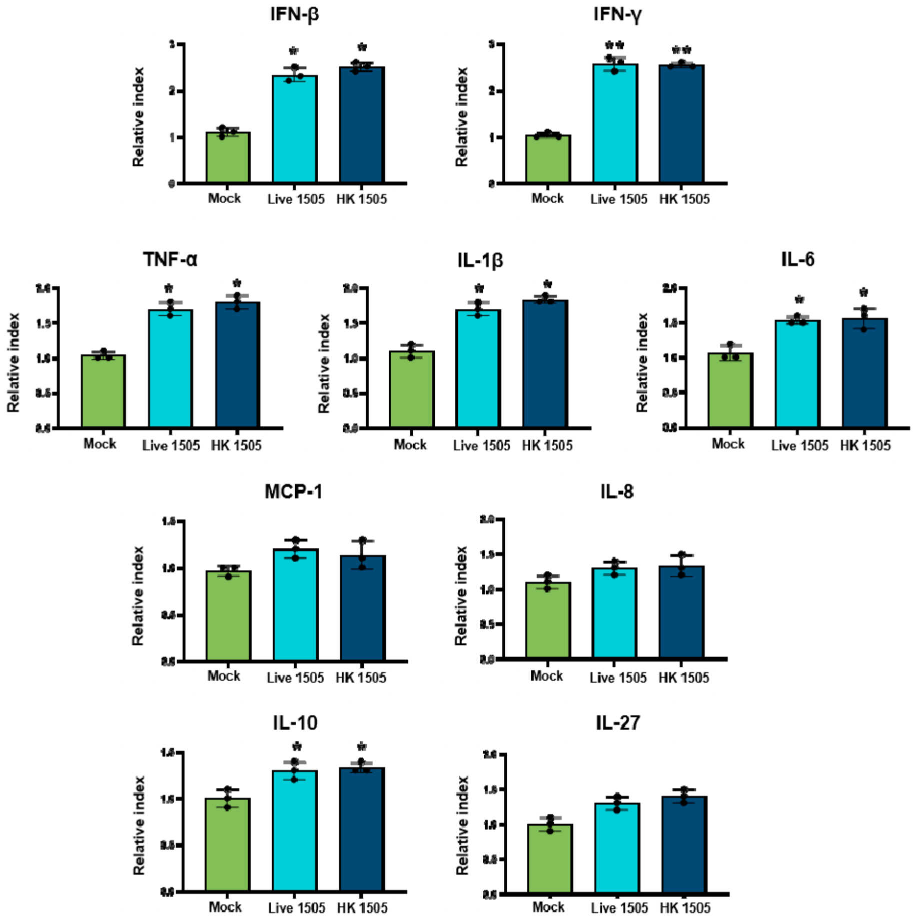


**Supplementary Figure S1.** Effect of *Lacticaseibacillus rhamnosus* CRL1505 on the expression of interferons and cytokines in porcine alveolar macrophages. Porcine alveolar macrophages (PAM cells) were stimulated with viable (Live 1505) or heat-killed (HK 1505) *L. rhamnosus* CRL1505 for 12 h (MOI=10). Non-lactobacilli-treated PAM cells (Mock) were used for comparisons. The expressions of interferons, proinflammatory cytokines and chemokines and regulatory cytokines were assessed using RT-qPCR. The results are shown as mean ± SD from three independent biological experiments (n = 3). Significant differences are shown compared to the control group at $p < 0.05$ (*) or $p < 0.01$ (**).

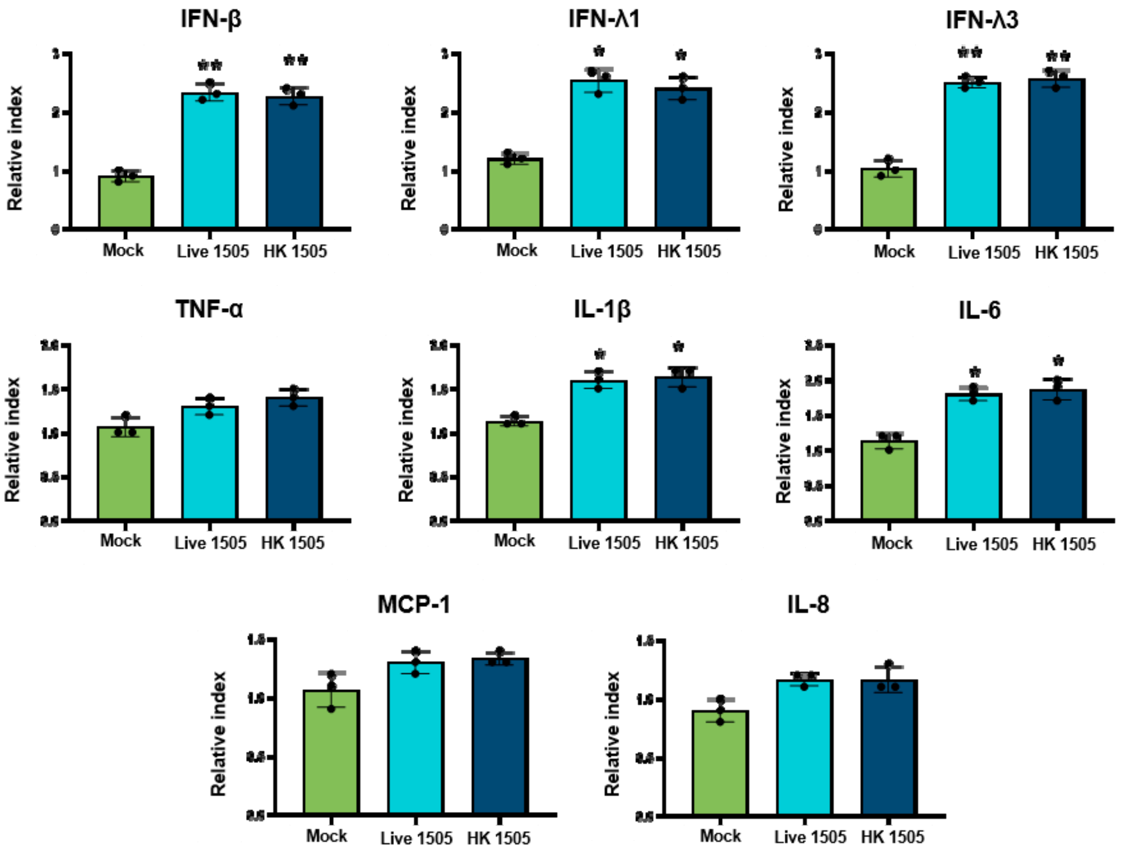


**Supplementary Figure S2.** Effect of *Lacticaseibacillus rhamnosus* CRL1505 on the expression of interferons and cytokines in porcine respiratory epithelial cells. Porcine bronchial epithelial cells (PBE cells) were stimulated with viable (Live 1505) or heat-killed (HK 1505) *L. rhamnosus* CRL1505 for 24 h (MOI=10). Non-lactobacilli-treated PBE cells (Mock) were used for comparisons. The expressions of interferons, proinflammatory cytokines and chemokines and regulatory cytokines were assessed using RT-qPCR. The results are shown as mean ± SD from three independent biological experiments (n = 3). Significant differences are shown compared to the control group at $p < 0.05$ (*) or $p < 0.01$ (**).
